# COVID-19 and the labour market: What are the working conditions in critical jobs?

**DOI:** 10.1186/s12651-022-00315-6

**Published:** 2022-07-26

**Authors:** Matthias Dütsch

**Affiliations:** Secretariat of the German Minimum Wage Commission, c/o Federal Institute for Occupational Safety and Health (BAuA), Nöldnerstraße 40-42, 10317 Berlin, Germany

**Keywords:** J81, J42, C31, COVID-19 pandemic, Critical jobs, Working conditions, Wages, Physical proximity, Working time patterns, Physical working conditions

## Abstract

The COVID-19 pandemic has focused public attention on occupational groups that ensure the maintenance of critical infrastructure, provision of medical care and supply of essential goods. This paper examines the working conditions in critical jobs based on representative data from the German BAuA Working Time Survey 2019. Our analyses reveal that essential workers are more likely to perform unskilled or semiskilled activities and work in cleaning, transport and logistics, health care occupations as well as IT and natural science services. Regarding the working conditions, essential workers are paid comparatively less and are more physically proximate to others at work than nonessential workers. They more often work atypical hours, such as day and night shifts and on weekends, and have less autonomy in their working time. Additionally, critical jobs are characterised by muscular and skeletal strain due to working positions and carrying heavy loads significantly more often. Thus, our findings strongly suggest that work-related risks accumulate in critical jobs.

**Supplementary Information:**

The online version contains supplementary material available at 10.1186/s12651-022-00315-6.

## Introduction

The COVID-19 pandemic has dramatically affected individuals’ social and economic lives. Many countries have put in place numerous requirements such as mask mandates and distancing measures and have communicated recommendations for avoiding social contact to protect against infection and contain the virus. In the implementation of these containment measures, the so-called critical economic sectors and critical occupations have become the focus of political and public attention. Government institutions worldwide drew up lists of critical sectors and occupations that are very similar in their composition; workers in these sectors ensure, among other things, the maintenance of systemically relevant infrastructure and the provision of medical care and nursing services and the supply of essential goods (see, e.g., CISA (2020) for the US, CPNI (2021) for the UK, and BMI (2009) for Germany). Unlike other employees who were asked to isolate themselves, work from home, and reduce their social contact at work, essential employees were provided with support measures, such as emergency child care, so that they could continue to perform their jobs.

Examining wages and physical proximity in critical occupations or industries, recent empirical research has indicated that the working conditions in critical jobs are less favourable than those in other jobs and have become even more hazardous during the pandemic. However, referring to theoretical approaches to segmented labour markets (Hendry 2003; Osterman 2011; Kaufman 2013) and research investigating the quality of work (Kalleberg 2011; Howell and Kalleberg 2019), we argue that it is necessary to investigate a broader range of working conditions since favourable and unfavourable working conditions are often found in a cumulative manner (ibid.). Beyond wages and physical proximity, working time patterns and physical working conditions are two additional crucial considerations. The former are important because working time arrangements influence everyday life (ILO 2018) and the organisation of work and family life (Howell and Kalleberg 2019). Both became even more significant when the measures to contain the COVID-19 pandemic were in effect since parents, particularly women, had to engage in child care and home schooling to a much greater extent than before. Unfavourable working hours also adversely affect individuals’ health. Furthermore, a wide range of studies have found physical working conditions to be important for individuals’ working lives. Physically demanding labour negatively influences health outcomes and leads to health inequalities and health-related job loss.

Against this backdrop, this paper raises the following research question: What are the working conditions in critical jobs? The study aims to perform a comprehensive analysis of working conditions in critical jobs and contributes to recent research in the following ways. First, we conceptually frame the public and academic discussion about working conditions in critical jobs by arguing that this debate can be linked to theoretical approaches to segmented labour markets. Second, we describe the sociodemographic characteristics of essential workers and structural determinants of critical jobs to enable policy decisions that protect and meet the needs of these workers. Third, we extend recent research on critical jobs by following Kalleberg’s (2011, p. 5) note that working conditions comprise multidimensional bundles of rewards and burdens. Therefore, we analyse wages, a classical dimension of inequality, and physical proximity to others at work, which we consider a new and emerging stressor due to the COVID-19 pandemic, as well as working time patterns and physical working conditions. Understanding which population strata are the most affected and gaining deeper insight into the working conditions in critical jobs is crucial for not only the persons concerned but also policymakers and stakeholders because research has examined the longer-term effects of past crises on labour market outcomes (Killewald and Zhuo 2019). Furthermore, employment-related exposure to SARS-CoV-2 endangers not only workers but also their household members (Selden and Berdahl 2020).

The empirical analyses are based on the German Federal Institute for Occupational Safety and Health (BAuA, for its German acronym) Working Time Survey 2019, conducted shortly before the beginning of the COVID-19 pandemic and therefore unaffected by it. The Working Time Survey is a representative study that includes detailed information on approximately 9,500 individuals from all industries. It is a unique dataset since it contains individual-level information on wages, physical proximity to others, working time patterns and physical working conditions, as well as the sociodemographic, job-related and structural characteristics of workers and their jobs. With reference to this dataset, employees' jobs were categorised as critical or noncritical based on the classification of systemically relevant supply and care occupations compiled by Burstedde et al. (2020) during the coronavirus pandemic in 2020. The list is based on the “List of Critical Infrastructures” (KRITIS) developed jointly by German federal states and the federal government in 2009; additionally, the classification of systemically relevant supply and care occupations includes occupations not yet covered by the KRITIS list but of particular relevance in the COVID-19 pandemic. This empirical approach allows us to examine the specific occupational strains that already existed before the COVID-19 pandemic in occupations that became very important during the pandemic. To our knowledge, there are no alternative and longitudinal data to draw representative conclusions about changes in working conditions due to the COVID-19 pandemic. The empirical investigation is conducted in three steps. First, we present a descriptive analysis of the data. Second, we perform binary logistic regressions to determine the likelihood of working in a critical job and, thus, identify the groups of employees concerned. Third, in various regression estimations, we investigate the relationships between critical jobs and working conditions to assess possible accumulation of risks. In doing so, we pursue an explorative approach and provide correlations since we cannot determine causal effects due to the cross-sectional data structure.

The paper is structured as follows. Section 2 describes the current state of research. Section 3 provides a theoretical rationale for the differences in working conditions between critical and noncritical jobs, while Sect. 4 presents the data and the methodological approach. Section 5 reports the empirical results, and Sect. 6 concludes.

## State of the research

Since the beginning of the pandemic, the literature on the groups of workers affected by the pandemic, as defined by their sociodemographic characteristics, and on the consequences of the COVID-19 emergency for individuals and households has been growing rapidly. However, to our knowledge, only a few research papers have focused on systemically relevant occupations.

Blau et al. (2020) studied the US labour market and drew on the federal guidelines of the Department of Homeland Security (DHS) and the Cybersecurity and Infrastructure Security Agency (CISA) to identify 194 out of 287 total NAICS industry categories as essential. Additionally, the study identified frontline workers as a subcategory of essential workers: those in occupational groups where a third of workers or fewer can feasibly work from home. They mapped both constructs to microdata from the 2017 and 2018 American Community Survey (ACS). Their descriptive comparison of the gender, race, educational degrees and hourly wages of essential and frontline workers revealed that the demographic and labour market characteristics of the broader group of essential workers tend to mirror their averages for all workers. In contrast, the narrower group of frontline workers is, on average, less educated, earns lower wages and is composed of more men, more individuals from disadvantaged minorities (especially groups of Hispanic ethnicity), and more immigrants. Kane and Tomer (2021) drew on the 4-digit NAICS industries related to the list of essential critical infrastructure workers and on employment data for each industry from the Bureau of Labor Statistics and defined a subgroup of frontline workers. Their descriptive results indicated that frontline workers earn lower wages and are more frequently required to be physically present in their workplace. Frontline workers also tend to be less educated than other essential workers and the wider US workforce. Essential workers are more often men working in construction, manufacturing, or skilled trades, while female employees in this group are much more concentrated in other essential occupations, such as health care, education, and service activities. Employing household data from the 2018 ACS, which is a random sample of US households, and the DHS list of essential critical infrastructure workers, McCormack et al. (2020) descriptively estimated that 25 percent of essential workers’ households are low income.

Looking at the German labour market, Koebe et al. (2020) investigated the social prestige and average wages associated with critical occupations. They used the German Socio-Economic Panel (GSOEP), a representative household survey, and classified “first-hour” and “second-hour” critical occupations based on the list issued by the federal state of Berlin. The list of first-hour critical occupations was published in Berlin on 17 March 2020 and was expanded approximately one month later to include the list of second-hour critical occupations.^1^ The data were operationalised at the 3-digit occupation classification level. By performing descriptive analyses, Koebe et al. (2020) found that essential employees are more likely to be women, to have below-average social prestige and to report below-average wages. These findings apply especially to first-hour critical occupations. Lübker and Zucco (2020) relied on German linked employer–employee data in their study and analysed employees in critical economic sectors. Applying logistic regressions, the authors revealed that women are more likely to work in critical infrastructure than men. This is also true for part-time workers and employees with technical jobs. In contrast, individuals without a university degree are slightly less likely to work in a critical sector. An assessment of the wages of full-time employees did not indicate systematic differences between critical and noncritical sectors.

The above review of literature on critical labour during the COVID-19 pandemic shows that many of the research papers have provided only descriptive evidence. The findings, whether for the US or for the German labour market, do not indicate clear patterns regarding the sociodemographics of essential employees. However, essential employees seem to earn comparatively low wages in poorly valued jobs and often perform work that requires greater physical proximity to others than nonessential work.

## Social inequalities, working conditions, and the COVID-19 pandemic

A theoretical rationale for the differences in working conditions between critical and noncritical jobs is lacking. Against the backdrop of recent studies that have identified apparently coinciding risks in critical jobs, we draw on newer theoretical approaches to human resource management that explain labour market segmentation (Hendry 2003; Osterman 2011; Kaufman 2013). These approaches assume imperfect labour markets and incomplete labour contracts. They emphasise that segmentation, and thus inequalities in the labour market, depend on employees’ and employers’ bargaining power and on the social and structural conditions that frame social actions within the employment system (ibid.).

Since employment relations are determined by the relative power of employers and employees to control tasks, negotiate the conditions of employment, and terminate employment, various aspects of job quality covary. If employers are interested in binding employees to the company for a longer period, they can achieve this through offering more secure and more highly paid jobs, better working conditions and further training opportunities. This creates closed positions in primary segments of the employment system (Hendry 2003; Osterman 2011; Kaufman 2013). In contrast, in more open and, therefore, secondary segments of the employment system, the problem of worker availability is quantitative only and is thus limited to the number of employees in external labour markets. Employees in open employment systems have little power of action in the labour market due to the competitive situation in their occupational field and the lack of representation of their interests. Therefore, this segment of the employment system is characterised by comparatively low wages and unfavourable noneconomic working conditions (ibid.). In fact, scholars could examine several individual, job-related and structural factors (such as gender, age, type of work, existence of work councils, firm size or economic sector) that are strongly associated with individuals’ positions in the primary or secondary labour market segments (Hudson 2007; Lucifora and Salverda 2009; Howell and Kalleberg 2019). Because working conditions are composed of multidimensional bundles of rewards and burdens (Muñoz de Bustillo et al. 2011; Kalleberg 2011), we consider four crucial components of working conditions in what follows.

First, the core dimension of job quality is certainly wages; wages are also regarded as the most straightforward attribute to measure (Muñoz de Bustillo et al. 2011; Howell and Kalleberg 2019). Wage inequality has been shown to be substantial and to have risen in many countries (Autor et al. 2008; Bol and Weeden 2015). Increased inequality across occupations and the associated heterogeneities across workplaces and firms (Card et al. 2013; Biewen et al. 2017) point to increased segmentation in the labour market. In terms of working conditions, sustained receipt of low wages is a serious issue because they have been shown to negatively influence, amongst other outcomes, individuals’ work satisfaction (Diaz-Serrano and Cabral 2005) and health (Kim and Leigh 2010; Leigh and Du 2012).

Second, as a result of the COVID-19 pandemic, epidemiological risk at work, considered to be a crucial component of working conditions, is not evenly distributed across workplaces and employees (Avdiu and Nayyar 2020; Basso et al. 2020; Dingel and Neiman 2020). Workers who are more highly exposed to aerosols due to a high degree of social interaction at work with customers, clients, and persons in need of care report deteriorated physical and mental health outcomes and face a greater risk of SARS-CoV-2 infection (Mhango et al. 2020; Sanghera et al. 2020). In contrast, in the case of home office work, work-related face-to-face interactions can be avoided, which reduces exposure to aerosols and therefore the risk of infection (Dingel and Neiman 2020).

Third, a further important aspect of working conditions is working time, as the ILO recently emphasised: “Working time, perhaps second only to wages, is the working condition that has the most direct impact on the day-to-day lives of workers” (ILO 2018, p 2). This dimension is especially relevant to the organisation of work and family life (Howell and Kalleberg 2019). Working time arrangements became even more significant when the measures to contain the COVID-19 pandemic were in effect since parents, particularly women, had to engage in child care and home schooling to a much greater extent than before (Alon et al. 2021). Furthermore, working time is crucial to employees’ health. Long working hours, including overtime hours, are negatively correlated with physical and psychological health (Bannai and Tamakoshi 2014; Kivimäki et al. 2015) and are positively correlated with the risk of workplace accidents (Dembe et al. 2005; Fischer et al. 2017). Regarding atypical working hours, studies have found negative health effects when work must be performed during socially valuable times—on Sundays, for example (Wirtz et al. 2011)—and particularly during night shifts (Costa 2003). Research has also provided evidence that a lack of job control over working hours, such as requirements to be on call or expectations to be accessible at all times, limits workers’ individual autonomy and places demands on employees, constituting stressors that negatively affect health (Väänänen et al. 2008; Slany et al. 2014). During the COVID-19 pandemic, the greatly increased work intensity was reported as a risk factor for the mental health of medical and nursing staff (Godderis et al. 2020; Sanghera et al. 2020).

Fourth, physical working conditions are another important aspect of job quality (Muñoz de Bustillo et al. 2011). Research has indicated that poor physical working conditions cause severe health problems (Laaksonen et al. 2010; Holtermann et al. 2011) and health inequalities (Kaikkonen and Rahkonen 2009) and lead to health-related job loss (Sewdas et al. 2019). Work that primarily requires the use of the musculoskeletal system to accomplish the corresponding tasks is described as physically demanding (de Kok et al. 2019). Such jobs include handling manual loads (such as lifting loads), working in forced postures (such as standing, sitting, or bending the torso), working with increased exertion, and completing highly repetitive manual tasks. Numerous systematic reviews have demonstrated the link between physical strain at work and musculoskeletal disorders, which are very common health problems (Holtermann et al. 2011). The prevalence of musculoskeletal disorders is associated with high levels of anxiety, sleeping problems and overall fatigue among workers; such disorders are also related to workers’ mental well-being (de Kok et al. 2019). Furthermore, physical stress causes, among other problems, cardiovascular diseases (Holtermann et al. 2011).

Against this backdrop, we focus on wages, physical proximity to others at work, working time patterns and physical working conditions to assess work-related risks in jobs crucial for the maintenance of social life during the pandemic. In the following section, we first describe the data, our operationalisation, and our method before presenting our empirical findings.

## Data and methodological approach

Our analyses are based on data from the BAuA Working Time Survey 2019, a nationally representative study of the German working population. The survey was designed and commissioned by the BAuA (Wöhrmann et al. 2021). Data from 9,382 individuals were collected in computer-assisted telephone interviews between May 2019 and January 2020—thus, *before* the COVID-19 pandemic hit Germany. This feature of the data is very important, as it ensures that respondents’ answers about their working conditions were unaffected by the COVID-19 pandemic; thus, unbiased estimates can be assumed. To be eligible to participate, individuals had to be 15 years of age or older and in paid employment for at least 10 h per week at the time of the interview. Employees who had interrupted their employment for longer than three months—for instance, because of maternity leave or periods of sickness—or who were engaged in vocational training or in military, civilian, or voluntary service were excluded. To compensate for survey-related selectivity and to ensure the representativeness of the data, the BAuA Working Time Survey provides weights to match the basic figures from the 2018 Microcensus of the Federal Statistical Office (Häring et al. 2020). The advantage of the Working Time Survey is that for the first time, all relevant information on monthly wages, hours worked, physical proximity to others at work, working time patterns and physical working conditions is available within a single dataset; it additionally enables a variety of sociodemographic and structural factors to be included and controlled for. The latter is particularly necessary because the cross-sectional data do not allow us to directly control for the possible selection of certain employees into certain (stressful) occupations. The inclusion of a rich set of covariates in our estimations should control for such selection effects to the greatest possible extent.

Based on the prepandemic data of the BAuA Working Time Survey 2019, to indicate whether an individual works in a critical job, we computed a dummy variable based on the classification of systemically relevant supply and care occupations compiled by Burstedde et al. (2020) during the coronavirus pandemic in 2020. This classification was developed in several steps. First, critical sectors were identified in the German Classification of Economic Activities (WZ 2008) based on the KRITIS list, which was developed jointly by German federal states and the federal government in 2009 (BMI 2009). In some cases, Burstedde et al. (2020) added sectors not (yet) included in the original KRITIS list but that became significant during the pandemic. Second, using data on employees subject to social insurance contributions by occupation and sector from the Federal Employment Agency (BA), Burstedde et al. (2020) identified occupations operating mainly in these sectors. For this purpose, they used the 1,286 occupational types from the 2010 German classification of occupations (KldB 2010).^2^ In most cases, this procedure led to a clear assignment of occupations to critical sectors. However, several occupations had to be examined individually and independently of the sector. To this end, Burstedde et al. (2020) relied on very detailed descriptions of 28,000 occupational titles (BA 2020a) and the BERUFENET database (BA 2020b) and assessed the extent to which the qualifications needed and tasks performed in an occupation were necessary for the production of supply-relevant goods and services or for public safety.^3^

The advantage of this fine-tuned identification of critical occupations based on the 1,286 occupational types is that the KRITIS list could be adapted as objectively as possible to the context of the COVID-19 pandemic. For example, trade was classified as relevant across the board in the original KRITIS list. However, under the COVID-19 pandemic, the sale of jewellery and watches and the music trade were by no means essential for critical infrastructures. In the food manufacturing sector, for example, occupations that produce alcoholic beverages were deemed not essential. In addition, some essential sectors such as waste disposal and funeral services were not (yet) enumerated on the federal government's KRITIS list but were explicitly listed in some federal state pandemic lists. This also applied to occupations in plastics and rubber manufacturing, which were needed during the pandemic for production of respirators and food packaging. Overall, this categorisation led to the delineation of 503 of the 1,286 occupational types as critical occupations. The list of critical occupations can be found in Burstedde et al. (2020: 27ff.) and in the appendix (Additional file 1: Appendix Table A1). Note, in general, that this occupational classification places special emphasis on the consideration of value chains; this implies that a larger number of occupations were defined as critical than under the narrower definitions of frontline work that have been the focus of public debates in the past (ibid.: 5).

Regarding the outcome variables, the Working Time Survey data allow us to calculate gross hourly wages based on gross monthly wages and weekly working hours. We obtained our figure for gross hourly wages by dividing gross monthly wages by weekly working time, which was multiplied by 4.33.^4^ Individuals who refused to answer the questions on wages and hours worked were dropped. These restrictions left us with a sample of 7,268 cases. We assessed the extent of physical proximity to others at work based on three questions in the Working Time Survey: “How often do you have direct contact at work with people or patients in need of care or assistance?”, “How often do you have direct contact at work with guests, customers or clients?” and “How often do you have direct contact at work with other people not employed by your employer?” Respondents could indicate whether such contact occurred often, sometimes, rarely or never. We created a dummy variable coded with the value 1 to indicate frequent physical proximity when at least one of the three questions above was answered with “often”. In all other cases, the value 0 was assigned, reflecting a work situation in which the employee is sometimes, seldom, or never in physical proximity to others at work. In recent research on physical proximity to others, home office work has been considered the exact opposite of proximity (Avdiu and Nayyar 2020; Dingel and Neiman 2020). Thus, we also included a home office indicator. Working time patterns are differentiated through measures of the duration of work, atypical work hours (weekly overtime, shift work, and weekend work) and working time autonomy (regularly being on call or standby, making one’s own decisions about breaks, being expected to be accessible in private life, and having the possibility to separate work and private life). Physical working conditions are measured by indicators for muscular and skeletal strain (working in a standing position; working in a sitting position; kneeling, bending, or engaging in overhead work; lifting and carrying heavy loads) and for strain from the working environment (noise; bright, poor, or faint light; cold, heat, wetness, dampness, or draughts; the inability to influence one’s work tasks).

In the first step, we assessed the determinants of working in a critical job with the following statistical model:

1 $$cjob={\alpha }_{0}+{\beta }_{1}{x}_{i}+\varepsilon ,$$

where $cjob$ is the dependent dummy variable (0 = noncritical job; 1 = critical job) following a binary logistic distribution $\left(P\left(y=1\right)=\frac{1}{1+{e}^{z}}\right)$. α is the regression constant, and β is the coefficient of the explanatory factors. The latter are added sequentially in three steps. Model 1 contains only sociodemographic (gender, age, place of residence, highest professional degree) and household (marital status, children in household) characteristics, model 2 adds job-related factors (tenure, form of employment, type of contract, job complexity, additional jobs), and model 3 includes structural determinants (size of company, work council) as well as occupational information (14 occupational segments). $\upvarepsilon$ denotes the error term.

In a second step, we investigated the correlation between employment in a critical job and working conditions. The formal statistical equation of the corresponding estimations is

2 $$z={\alpha }_{0}+{\beta }_{1}cjob+{\gamma }_{i}{x}_{i}+\varepsilon ,$$

where z denotes the dependent variable. α is the regression constant, and β is the coefficient of interest indicating the correlation with employment in a critical job. $\upgamma$ reflects the influence of the other covariates, and $\upvarepsilon$ is the error term. Hourly wages are logarithmised and estimated using a linear Mincerian regression. The two indicators of physical proximity are binary coded and follow a binary logistic distribution $\left(P\left(y=1\right)=\frac{1}{1+{e}^{z}}\right)$. Weekly overtime (in hours) is subject to a linear regression, while the variables on atypical working hours and weekend work adhere to a multinomial logistic function $\left(\mathrm{P}\left(y=J\right)=\frac{1}{1+\sum_{j=1}^{J-1}{e}^{z}}\right)$ with $\mathrm{J}$ categories of the variable. Regarding working time autonomy, the indicators of “Regular on-call or standby service”, “Make own decisions about breaks” and “Separation of work and private life possible” are subject to a binary logistic distribution, and the indicator of accessibility in private life is subject to a multinomial logistic function. All variables regarding “Muscular and skeletal strain” and “Strain from the working environment” are binary coded and accordingly follow a binary logistic distribution. To take into account that the assignment of employees to critical and noncritical jobs might not happen randomly, we obtained cluster-robust standard errors for 144 occupational groups of the German Classification of Occupations 2010 from the regression analyses.

To check the robustness of our results regarding the categorisation of critical jobs, we re-estimated our analyses with two different classifications (see Sect. 5.4), both of which have been used in empirical research investigating critical jobs in Germany. The first one of Koebe et al. (2020) comprises the list of essential frontline workers of the federal state of Berlin compiled at the onset of the coronavirus pandemic at the less differentiated three-digit level of the classification of occupations. The second re-estimation was carried out on the basis of the 88 divisions of the German Classification of Economic Activities 2008 (Federal Statistical Office 2008) and represents the original KRITIS list without coronavirus-conditional modifications. The KRITIS list was the starting point for all classifications of critical jobs and has been applied to analyses of the corona pandemic by Lübker and Zucco (2020). This list is comparatively narrowly defined and includes, in particular, sectors and occupations needed in the short term to provide basic services to the population. This operationalisation of jobs as critical can be found in Pfeiffer (2020: 68; fifth column).

## Results

### Descriptive results

**Fig. 1:**
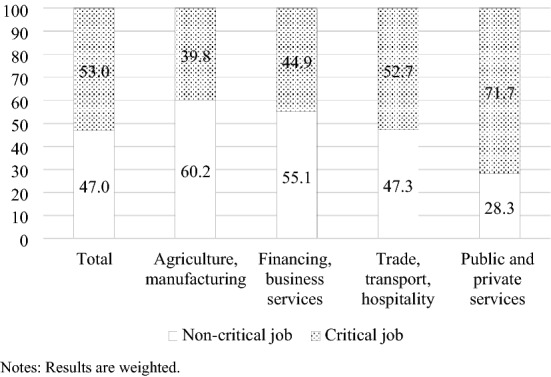
Share of critical jobs (in percent). Source: Working Time Survey 2019; own calculations

Among the 31.8 million employees in our analysis sample, 17 million, or 53%, work in a critical job (Fig. 1). Furthermore, critical jobs are found to varying degrees in the different sectors of the economy. Such jobs comprise comparatively small shares of the agriculture and manufacturing (39.8%) and financing and business services (44.9%) sectors. A total of 52.7% of critical jobs are observed in the trade, transport and hospitality sector; the highest share of critical jobs is in the public and private services sector (71.7%).

On average, employees in Germany earn 19.52 euros (Table 1). Employees in critical jobs are paid 18.74 euros per hour, slightly less than those in noncritical jobs, as the latter are paid 20.19 euros per hour. Among the ten lowest-paid critical occupations are cleaning services, (retail) sales occupations selling foodstuffs and doctors’ receptionists and assistants (Table 2).

**Table 1:** Descriptive statistics on wages, physical proximity, working time patterns and physical working conditions

		All observations		Critical workers		Noncritical workers	
		Mean	SD	Mean	SD	Mean	SD
Wages	Hourly wage	19.52	11.469	18.74	11.752	20.19	11.178
Physical proximity at work	Physical proximity to others	0.76	1.317	0.85	1.280	0.68	1.342
	Home office work	0.20	0.693	0.12	0.583	0.26	0.769
Duration of work and atypical working hours	Weekly overtime (in hours)	3.23	4.161	3.33	4.496	3.14	3.849
	Working hours usually between 07:00 and 19:00	0.71	0.455	0.63	0.482	0.77	0.420
	Only early or late shift work: working hours not between 07:00 and 19:00	0.10	0.304	0.11	0.317	0.09	0.291
	Shift work without night work	0.10	0.299	0.12	0.323	0.08	0.276
	Shift work and night work	0.09	0.287	0.14	0.343	0.05	0.223
	No weekend work	0.60	0.491	0.51	0.500	0.67	0.470
	Work on Saturday	0.17	0.380	0.17	0.372	0.18	0.385
	Work on Saturday and Sunday	0.23	0.420	0.33	0.469	0.15	0.355
Working time autonomy	Regular on-call or standby service	0.09	0.293	0.13	0.342	0.06	0.238
	Make own decisions about breaks	0.34	0.641	0.30	0.649	0.37	0.631
	Not expected to be accessible in private life	0.63	0.483	0.60	0.491	0.66	0.475
	Expected to be partially accessible in private life	0.15	0.354	0.15	0.357	0.14	0.351
	Expected to be accessible in private life	0.22	0.417	0.25	0.435	0.20	0.400
	Separation of work and private life possible	0.73	0.453	0.69	0.461	0.77	0.444
Muscular and skeletal strain	Working in a standing position	0.53	0.509	0.63	0.483	0.45	0.515
	Working in a sitting position	0.55	0.507	0.51	0.521	0.59	0.491
	Kneeling, bending, working over head	0.16	0.368	0.19	0.389	0.14	0.348
	Lifting and carrying heavy loads	0.20	0.403	0.26	0.439	0.16	0.363
Strain from the working environment	Noise	0.30	0.468	0.32	0.490	0.28	0.448
	Bright, poor, faint light	0.12	0.351	0.15	0.413	0.09	0.283
	Cold, heat, wetness, dampness, draughts	0.24	0.459	0.29	0.497	0.20	0.419
	Can influence the work tasks that must be carried out	0.35	0.587	0.31	0.585	0.38	0.587

Results are weighted

Source: Working Time Survey 2019; own calculations

**Table 2:** Critical occupations with the lowest hourly wages

Occupations	Average hourly wage
Occupations in the production of clothing and other textile products	6.24
Occupations in gardening	8.77
Occupations in cleaning services	9.63
Technical occupations in railway, aircraft and ship operation	11.08
Cooking occupations	12.30
Sales occupations (retail) selling foodstuffs	12.50
Driver of vehicles in road traffic	13.04
Occupations in animal husbandry	13.34
Doctors’ receptionists and assistants	13.39
Drivers and operators of construction and transportation vehicles and equipment	13.39

Results are weighted

Source: Working Time Survey 2019; own calculations

Furthermore, Table 1 shows that approximately 76% of all jobs involve physically proximate activities. Compared to employees in noncritical jobs, those engaged in critical jobs are 17 percentage points more likely to work physically proximately to others. Home office work, which ensures distance from others while working, can be performed by 20% of employees; however, this proportion is significantly lower for essential employees (12%) than for nonessential employees (26%). Regarding the duration of work, weekly overtime is slightly higher among those in critical jobs (3.33 h compared to 3.14 h). Shift work and night work represent atypical working hours. In this respect, normal working hours during the day (between 07:00 and 19:00) are less common in critical jobs (63%) than in noncritical jobs (77%); however, rotating shifts without and with night work are approximately 4 and 9 percentage points more common in critical jobs, respectively. There are also crucial between-group differences with regard to weekend work. Working on both Saturdays and Sundays is much more common among essential employees (33%) than among nonessential employees (15%). Working time autonomy is lower among essential employees because they are more regularly on call or on standby than are nonessential employees (13% compared to 6%) and are less able to make decisions about their breaks themselves (37% compared to 30%). In addition, essential employees are expected to be accessible for work-related matters in their private lives more often. Regarding muscular and skeletal strain, essential employees more often perform their work in a standing position (63% compared to 45%) or in a kneeling, bending, or overhead position (19% compared to 14%) and must lift and carry heavy loads more often than other workers (26% compared to 16%). Strain from the working environment, such as working under bright, poor or faint light or in cold, hot, wet, damp or draughty conditions, is more frequently reported by essential employees (15% and 29%) than by other employees (9% and 20%).

Given the great importance of critical jobs for the economy, first, the sociodemographic, job-related and structural determinants of employment in critical jobs are assessed; second, the working conditions in those jobs are examined.

### Determinants of working in a critical occupation

The probability of performing a critical job, presented in Table 3, is determined based on three estimations, into which the explanatory variables are added sequentially.^5^ Model 1 contains sociodemographic and household characteristics, model 2 adds job-related factors, and model 3 includes structural determinants as well as occupational information. A comparison across the results of models 1, 2 and 3 reveals that the inclusion of the additional variable blocks in models 2 and 3 causes some significant correlations with sociodemographic and household characteristics to become insignificant.

**Table 3:** Determinants of working in a critical job (logistic regressions)

	(1)	(2)	(3)
	Critical job (AME)	Critical job (AME)	Critical job (AME)
Gender (1 = female)	0.321^***^ (0.048)	0.142^*^ (0.057)	0.080 (0.072)
Age (in years)	− 0.006 (0.020)	− 0.009 (0.021)	− 0.006 (0.025)
Age squared (in years)	0.000 (0.000)	0.000 (0.000)	− 0.000 (0.000)
Place of residence (1 = East Germany)	0.067^**^ (0.026)	0.074^**^ (0.028)	0.079^*^ (0.032)
Highest professional degree (Ref.: University degree)			
Vocational degree	0.202^***^ (0.058)	− 0.219^**^ (0.083)	− 0.180 (0.100)
Technical school, master	0.172^*^ (0.075)	− 0.058 (0.091)	− 0.020 (0.111)
Polytechnic degree	0.185^*^ (0.081)	0.011 (0.087)	0.185 (0.098)
Another degree	0.420 (0.219)	0.294 (0.236)	0.402 (0.300)
No professional degree	0.209 (0.166)	− 0.392^*^ (0.187)	− 0.551^*^ (0.243)
Unknown	0.285 (0.460)	− 0.012 (0.504)	− 0.158 (0.509)
Marital status (Ref.: single)			
Married	− 0.061 (0.068)	− 0.093 (0.070)	0.402 (0.300)
Civil union	0.067 (0.241)	0.098 (0.253)	− 0.551^*^ (0.243)
Divorced/widowed	0.077 (0.087)	0.092 (0.089)	− 0.158 (0.509)
Unknown	1.637 (1.046)	1.805 (1.037)	0.402 (0.300)
Children in the household (Ref: no children in the household)			
Child younger than 7 years in the household	0.019 (0.090)	0.006 (0.094)	− 0.055 (0.111)
Child aged 7 to 12 years in the household	0.178^*^ (0.087)	0.147 (0.090)	0.085 (0.105)
Child aged 13 to 18 years in the household	0.161 (0.084)	0.140 (0.086)	0.140 (0.103)
Tenure (in years)		0.007^**^ (0.002)	0.009^**^ (0.003)
Form of employment (Ref.: full-time)			
Part-time		0.255^***^ (0.066)	0.107 (0.077)
Marginal employment		− 0.264 (0.246)	− 0.716^*^ (0.324)
Unknown		− 1.402^*^ (0.615)	− 2.200^**^ (0.697)
Type of contract (1 = permanent contract)			
Fixed-term contract		− 0.077 (0.107)	− 0.203 (0.127)
Unknown		1.205^***^ (0.094)	1.063^***^ (0.110)
Complexity of job (Ref.: unskilled or semi-skilled activity)			
Specialist activity		− 0.853^***^ (0.148)	− 0.142 (0.189)
Complex specialist activity		− 1.295^***^ (0.156)	− 0.866^***^ (0.199)
Highly complex activity		− 1.678^***^ (0.162)	− 1.456^***^ (0.206)
Additional jobs (Ref.: no additional job)			
One additional job		− 0.085 (0.098)	− 0.255^*^ (0.120)
More than one additional job		− 0.447 (0.246)	− 0.417 (0.312)
Size of company (Ref.: more than 500 employees)			
Fewer than 9 employees			− 0.089 (0.130)
10–49 employees			0.204^*^ (0.092)
50–499 employees			0.048 (0.076)
Unknown			0.107 (0.276)
Work council (Ref.: existent)			
Nonexistent			− 0.428^***^ (0.080)
Unknown			0.067 (0.196)
Occupational segments (Ref.: manufacturing)			
Agriculture, forestry and gardening			0.670^*^(0.301)
Manufacturing engineering			1.219^***^ (0.177)
Construction			1.295^***^ (0.191)
Food and hospitality			1.803^***^ (0.227)
Medical and nonmedical health care			4.273^***^ (0.218)
Social and cultural services			2.398^***^ (0.184)
Retail and trade			0.700^***^ (0.191)
Corporate management and organisation			− 0.580^**^ (0.206)
Business services			1.775^***^ (0.176)
IT and natural science services			3.000^***^ (0.194)
Security			2.924^***^ (0.273)
Transport and logistics			5.324^***^ (0.351)
Cleaning			5.462^***^ (1.053)
Number of observations	7,268	7,268	7,268
Pseudo R^2^		0.048	0.278

The table shows the estimates obtained from the regression model indicated in Eq. (1). AMEs are the average marginal effects. Cluster-robust standard errors for 144 occupational groups in parentheses; *p < 0.05, **p < 0.01, ***p < 0.001

Source: Working Time Survey 2019; own calculations

According to model 3, there are no gender differences in the probability of working in a critical job. While there is no statistically significant correlation with age, East German workers are more often observed in critical jobs. The results indicate a lower probability of being employed in a critical job for workers without a vocational degree. Overall, the results on household characteristics do not display significant correlations.

Regarding job-related factors, it is evident that the probability of being an essential employee rises with increasing tenure. The opposite is the case for workers in marginal employment. While the type of contract does not have any significant influence on this probability, the complexity of the job plays a role. In particular, employees who perform complex specialist activities or highly complex activities work in critical jobs significantly less often than employees in unskilled or semiskilled activities. The latter also applies to employees who have an additional job. Regarding structural factors, we find that critical jobs are performed more frequently in medium-sized companies (those with between 10 and 49 employees). Employees in companies that do not have a work council are less likely to engage in critical jobs. Relative to the probability in the occupational segment of manufacturing, the highest probabilities of working in a critical job exist in the cleaning, transport and logistics, medical and nonmedical health care and IT and natural science services segments.

### Working conditions in critical jobs

To identify risk factors in critical jobs, the following analyses examine wages, physical proximity to others at work, working time patterns and physical working conditions. Due to the different scales of the dependent variables, we estimate various multiple regressions. The corresponding functional form—linear, binary logistic or multinomial logistic estimation—is indicated in the tables.

The first crucial dimension of working conditions is wages. We perform Mincerian regressions on logarithmically transformed hourly wages and control for sociodemographic, job-related and structural factors.^6^ The central variable of interest, the dummy indicator for whether a job is critical or not, is significantly negative (Table 4). This coefficient implies that essential employees earn 2.08% lower wages than nonessential employees.^7^

**Table 4:** Estimation of wages (OLS regression)

	Hourly wages (log) (Coef.)
Critical job (1 = yes)	− 0.021* (0.009)
Sociodemographic characteristics	x
Job characteristics	x
Structural characteristics	x
Number of observations	7,268
*R*^2^	0.424

The table shows the estimates obtained from the regression model indicated in Eq. (2). The estimation also includes the sociodemographic, job-related and structural characteristics (without occupational segments) presented in model 3 of Table 3 as control variables. Cluster-robust standard errors for 144 occupational groups in parentheses; *p < 0.05, **p < 0.01, ***p < 0.001

Source: Working Time Survey 2019; own calculations

The second important dimension of working conditions during the COVID-19 pandemic is the degree of physical proximity to others at work (Table 5). Employees in critical jobs have a 13.2 percentage-point higher probability of performing a physically proximate job than nonessential workers. They are, on the other hand, 6.2 percentage points less likely to have the opportunity to work from home.

**Table 5:** Estimation of physical proximity to others at work

	Binary logistic regression	Binary logistic regression
	Physical proximity (AME)	Home office work (AME)
Critical job (1 = yes)	0.132*** (0.011)	− 0.062*** (0.009)
Sociodemographic characteristics	x	x
Job characteristics	x	x
Structural characteristics	x	x
Number of observations	7,268	7,251
Psabeudo R^2^	0.058	0.175

The table shows the estimates obtained from the regression model indicated in Eq. (2). The estimations also include the sociodemographic, job-related and structural characteristics (without occupational segments) that are presented in model 3 of Table 3 as control variables. AMEs are the average marginal effects. Cluster-robust standard errors for 144 occupational groups in parentheses; *p < 0.05, **p < 0.01, ***p < 0.001

Source: Working Time Survey 2019; own calculations

The third dimension of working conditions is working time patterns. Table 6 presents the results for the duration of work and atypical working hours. Employees in critical jobs work overtime significantly more often. In addition, essential employees have a higher probability of working early or late shifts, rotating day shifts and shift and night work. With regard to weekend work, there are no differences in the probability of working on Saturdays; however, essential employees are more likely to work on Sundays.

**Table 6:** Estimation of working time patterns—duration of work and atypical working hours

	Linear regression (OLS)	Multinomial logistic regression (Base outcome: Working hours usually between 07:00 and 19:00)			Multinomial logistic regression (Base outcome: No weekend work)	
	Weekly overtime (in hours) (Coef.)	Only early or late shift work (AME)	Shift work without night work ( AME)	Shift work and night work (AME)	Work on Saturdays (AME)	Working on Saturdays and Sundays (AME)
Critical job (1 = yes)	0.493*** (0.109)	0.024*** (0.007)	0.025*** (0.006)	0.034*** (0.006)	0.005 (0.009)	0.115*** (0.010)
Sociodemographic characteristics	x	x	x	x	x	x
Job characteristics	x	x	x	x	x	x
Structural characteristics	x	x	x	x	x	x
Number of observations	7,268	7,214	7,214	7,214	6,934	6,934
*R*^2^/Pseudo R^2^	0.081	0.212	0.212	0.212	0.131	0.131

The table shows the estimates obtained from the regression model indicated in Eq. (2). The estimations also include the sociodemographic, job-related and structural characteristics (without occupational segments) presented in model 3 of Table 3 as control variables. AMEs are the average marginal effects. Cluster-robust standard errors for 144 occupational groups in parentheses; *p < 0.05, **p < 0.01, ***p < 0.001

Source: Working Time Survey 2019; own calculations

Disadvantageous job characteristics are also apparent when we consider working time autonomy (Table 7). Critical jobs are more likely to be associated with regular on-call or standby service. Furthermore, essential employees report being able to decide on their breaks by themselves comparatively less often than other workers. The expectations of superiors and colleagues that workers are accessible in their private lives are higher in critical jobs. Finally, essential employees have a lower probability of finding it possible to separate work and private life.

**Table 7:** Estimation of working time patterns—working time autonomy

	Binary logistic regression	Binary logistic regression	Multinomial logistic regression (Base outcome: Not expected to be accessible in private life)		Binary logistic regression
	Regular on-call or standby service (AME)	Make own decisions about breaks (AME)	Partially expected to be accessible in private life (AME)	Expected to be accessible in private life (AME)	Separation of work and private life possible (AME)
Critical job (1 = yes)	0.086*** (0.008)	− 0.032** (0.011)	0.010 (0.008)	0.040*** (0.010)	− 0.036*** (0.011)
Sociodemographic characteristics	x	x	x	x	x
Job characteristics	x	x	x	x	x
Structural characteristics	x	x	x	x	x
Number of observations	7,240	7,247	7,263	7,263	7,266
Pseudo R^2^	0.112	0.048	0.035	0.035	0.035

The table shows the estimates obtained from the regression model indicated in Eq. (2). The estimations also include the sociodemographic, job-related and structural characteristics (without occupational segments) presented in model 3 of Table 3 as control variables. AMEs are the average marginal effects. Cluster-robust standard errors for 144 occupational groups in parentheses; *p < 0.05, **p < 0.01, ***p < 0.001

Source: Working Time Survey 2019; own calculations

The last dimension examined concerns physical working conditions. With regard to muscular and skeletal strain, critical jobs are performed in a standing position significantly more often but in a sitting position less frequently than other jobs (Table 8). Additionally, essential employees work more often in a kneeling or bending position or above their heads. They also have to lift and carry heavy loads more frequently.

**Table 8:** Estimation of physical working conditions—muscular and skeletal strain

	Binary logistic regression	Binary logistic regression	Binary logistic regression	Binary logistic regression
	Working in a standing position (AME)	Working in a sitting position (AME)	Kneeling, bending, or overhead work (AME)	Lifting and carrying heavy loads (AME)
Critical job (1 = yes)	0.109*** (0.011)	− 0.068*** (0.010)	0.050*** (0.007)	0.061*** (0.008)
Sociodemographic characteristics	x	x	x	x
Job characteristics	x	x	x	x
Structural characteristics	x	x	x	x
Number of observations	7,268	7,268	7,252	7,268
Pseudo R^2^	0.278	0.269	0.264	0.247

The table shows the estimates obtained from the regression model indicated in Eq. (2). The estimations also include the sociodemographic, job-related and structural characteristics (without occupational segments) presented in model 3 of Table 3 as control variables. AMEs are the average marginal effects. Cluster-robust standard errors for 144 occupational groups in parentheses; *p < 0.05, **p < 0.01, ***p < 0.001

Source: Working Time Survey 2019; own calculations

Concerning strain from the working environment, essential employees report significantly more often that they do their job in noisy conditions; in bright, poor or faint light; and in cold, hot, wet, damp or draughty conditions (Table 9). Moreover, they can less frequently influence the work tasks that must be carried out than their nonessential counterparts.

**Table 9:** Estimation of physical working conditions—strain from the working environment

	Binary logistic regression	Binary logistic regression	Binary logistic regression	Binary logistic regression
	Noise (AME)	Bright, poor, or faint light (AME)	Cold, heat, wetness, dampness, or draughts (AME)	Can influence the work tasks that must be carried out (AME)
Critical job (1 = yes)	0.045*** (0.010)	0.027*** (0.007)	0.043*** (0.009)	− 0.013 (0.011)
Sociodemographic characteristics	x	x	x	x
Job characteristics	x	x	x	x
Structural characteristics	x	x	x	x
Number of observations	7,261	7,268	7,252	7,246
Pseudo R^2^	0.187	0.097	0.217	0.047

The table shows the estimates obtained from the regression model indicated in Eq. (2). The estimations also include the sociodemographic, job-related and structural characteristics (without occupational segments) presented in model 3 of Table 3 as control variables. AMEs are the average marginal effects. Cluster-robust standard errors for 144 occupational groups in parentheses; *p < 0.05, **p < 0.01, ***p < 0.001

Source: Working Time Survey 2019; own calculations

### Robustness checks

To check the robustness of our results regarding the categorisation of critical jobs, we re-estimated the determinants of critical occupations and their working conditions in two ways. First, we used the alternative three-digit level classification of first-hour occupations from Koebe et al. (2020), which focuses more narrowly on frontline work.^8^ Second, we employed the operationalisation of critical sectors from the original KRITIS list without the COVID-19-conditional modifications, which is also comparatively narrowly defined (Pfeiffer 2020: 68).^9^

The basic descriptive results reveal marked differences. While the classification of Burstedde et al. (2020) identifies 53.48% of jobs as critical, the figures are considerably smaller when we use the three-digit classification of Koebe et al. (2020) (41.3%) or the original KRITIS list (40.9%). This indicates that the classification of Burstedde et al. (2020) identifies more than just frontline workers and considers the value chains more broadly than the KRITIS list, which specifies a limited number of sectors and occupations needed in the short term to provide basic services to the population.

The robustness tests for the determinants of working in a critical job accordingly show differences associated with the use of both the narrower definitions of critical labour (Additional file 1: Appendix Table A3). The frontline work categorisation of Koebe et al. (2020) is characterised more significantly by female employment, younger workers and those with a technical school or master’s degree. Part-time workers, those performing a specialist activity and those holding an additional job work more often in critical jobs.^10^ In contrast, the KRITIS classification points to a lower probability of carrying out a critical job for workers in East Germany and those with a vocational, technical school or master’s degree. This also applies to employees who have children. The form of employment, holding an additional job, the size of the company or the existence of a work council have no impact. The results of the robustness checks on working conditions in critical jobs (Additional file 1: Appendix Table A3) based on the classification by Koebe et al. (2020) differ from our main results and become nonsignificant regarding weekly overtime, early or late shift work, the expectation of superiors and colleagues that workers are accessible in their private lives, the separation of work and private life and most variables related to physical strain from the working environment. Under the KRITIS operationalisation, almost none of the indicators of wages, physical proximity to others at work, duration of work and atypical work hours, or working time autonomy are related to employment in a critical job. Overall, the robustness checks for the determinants of working in a critical job indicate that the findings of which worker characteristics are determinant depend on the group definition used.

Regarding working conditions (Additional file 1: Appendix Table A4), use of the two coronavirus-specific classifications from Burstedde et al. (2020) and Koebe et al. (2020) reveal disadvantages in terms of wages and higher physical proximity to others in critical jobs. Such jobs are also associated with more atypical working hours and less working time autonomy. Workers in such jobs are more affected by muscular, skeletal, and environmental strains from their working positions, carrying of heavy loads and exposure to noise levels. However, under the KRITIS classification, which focuses on basic services to the population and does not include sectors or occupations of major importance during the COVID-19 pandemic, the results are very strongly divergent. This suggests that the KRITIS list is of only limited validity for studies on the COVID-19 pandemic.

## Discussion of results

The COVID-19 pandemic has had a strong impact on various dimensions of social inequality in the labour market and on work-related strains. This seems to be particularly the case for employees in systemically relevant occupations (Blau et al. 2020; Lübker and Zucco 2020; Koebe et al. 2020) that ensure the maintenance of critical infrastructure and the provision of medical care and nursing services and the supply of essential goods. Such employees were asked by political actors and the general public to continue working despite the health risks arising from the pandemic. These special circumstances increased the public’s awareness of essential occupational groups and raised questions surrounding the conditions under which essential employees work. However, only three quantitative analyses on this topic have been available to date (Blau et al. 2020; Lübker and Zucco 2020; Koebe et al. 2020) and have provided inconsistent results regarding working conditions in critical jobs due to data restrictions and the analyses’ mainly descriptive character. Against this backdrop, this study performed a more comprehensive analysis of working conditions in critical jobs. We were able to expand on previous research in three ways.

First, regarding data and methods, we used the representative German Working Time Survey 2019 to conduct our empirical analyses. These data allowed us both to identify critical jobs and to examine four dimensions of working conditions. The data were collected before the COVID-19 pandemic hit Germany, which ensures that the comparison groups and the variables of interest were unaffected by the pandemic. Furthermore, jobs could be classified at the 5-digit occupational level based on the classification of Burstedde et al. (2020), which made possible a differentiated categorisation of occupations by their systemic relevance during the COVID-19 pandemic. Our rich dataset allowed us to move beyond the descriptive evaluations that have predominated to date, identify working conditions through multiple estimations, and control for a variety of sociodemographic, job-related and structural factors.

Second, in terms of content, our descriptive findings indicated that 53.48% of employees in the survey sample worked in a critical job. Our multiple regressions revealed that employees living in East Germany and those with longer job tenure more often worked in critical jobs. Critical jobs were more often located in medium-sized companies and in companies with a work council. Furthermore, critical jobs could be found in the cleaning, transport and logistics, medical and nonmedical health care and IT and natural science services segments, a finding in line with those of Blau et al. (2020) and Koebe et al. (2020).

Third, regarding working conditions, our descriptive analyses showed that essential employees earned on average 18.74 euros per hour (gross) and thus 1.45 euros less than other employees. Among the lowest paid critical occupations were those in cleaning services, (retail) sales occupations selling foodstuffs and doctors’ receptionists and assistants. The multiple estimates confirmed our descriptive findings and the previous findings of Blau et al. (2020) and Koebe et al. (2020) that essential workers receive lower wages. Furthermore, essential employees were 13.2 percentage points more likely to work in jobs requiring physical proximity to others and could do home office work significantly less often. Both findings accord with recent research on the correlation between critical work and physical proximity (Avdiu and Nayyar 2020; Dingel and Neiman 2020). Concerning working time patterns, critical jobs were associated with overtime work and atypical working hours (day and night shifts and Sunday work) significantly more often than other jobs and involved a lesser degree of working time autonomy due to requirements to regularly be on call or standby, higher expectations for accessibility in private life, fewer opportunities to make decisions about breaks and an impossibility of separating their work and private life. With regard to physical working conditions, our estimates indicated exposure to greater muscular and skeletal strain in critical jobs because workers had to work more frequently in a standing, kneeling or bending position or in overhead activities and because of the requirement to lift and carry heavy loads. Finally, we revealed greater strain from the working environment (noisy conditions; bright, poor or faint light; cold, hot, wet, damp or draughty conditions; and the inability to influence one’s work tasks) in critical jobs.

Fourth, the theoretically basis of our research on systemically relevant jobs referred to approaches to human resource management that explain labour market segmentation (Hendry 2003; Osterman 2011; Kaufman 2013). According to these approaches, employees are particularly likely to occupy unfavourable positions in the labour market when they have little power to act, which can be explained by access to resources such as professional knowledge and skills or by the specificity of their learned profession, legal regulations, collective agreements, or internal institutions such as work councils (ibid.). While we did not discover educational differences between essential and nonessential employees (the former even had longer work tenures), we did observe higher probabilities of working in critical jobs among employees performing unskilled or semiskilled activity, who have also been found to occupy inferior positions in the employment system in other research (Lucifora and Salverda 2009; Howell and Kalleberg 2019). Furthermore, essential employees reported a comparatively higher prevalence of work councils. Thus, except for the distribution of workers carrying out unskilled or semiskilled activity, the sociodemographic and structural determinants of interest in our research did not reflect the crucial characteristics of employment in unfavourable labour market positions. However, a closer look at the occupational segments indicates that critical jobs are often located in sectors with little or no collective bargaining coverage, such as security, cleaning, transport and logistics and retail and trade (Ellguth and Kohaut 2019). Finally, our findings on working conditions align with research on segmented labour markets (Kalleberg 2011; Osterman 2011; Kaufman 2013). In fact, the risks of significantly lower wages, higher physical proximity to others at work, longer working hours, more atypical working hours, less working time autonomy and greater muscular and skeletal strain and strain from the working environment tend to accumulate in critical jobs.

## Conclusions

The COVID-19 pandemic has focused public attention on occupational groups that are highly important to the functioning of social life. Our empirical analyses highlighted that risks resulting from working conditions in critical jobs do not occur separately but cumulatively, which leads to severe health risks, as the cited literature has revealed. This accumulation of risks already characterised such jobs before the pandemic. However, these unfavourable working conditions were exacerbated by the fact that the pandemic has aggravated existing strains.

A possible beneficial federal measure would be to define the group of critical jobs more precisely. As our robustness checks showed, the sociodemographic, job-related and structural characteristics related to critical jobs changed according to the definition of critical jobs used. A formal list based on common industry codes or occupational classifications could be used to better prioritise safety measures, provision of protective equipment and other targeted benefits.

A further and already well-known public policy implication is related to occupational wage inequality. Our findings indicated that critical jobs are predominantly low-paid occupations in sectors with low collective bargaining coverage. Therefore, a longer-term measure would be to increase collective bargaining coverage in these sectors of the economy to raise the attractiveness of critical jobs. Because simply showing up to work has put many essential workers at risk, the high physical proximity to others and the associated risk of infection make it necessary to provide frequent COVID-19 tests and to cover hospitalisation and health costs.

Work-related strains from long and atypical working hours and physically demanding work increased during the COVID-19 pandemic since the labour of essential employees was required on a larger scale and with greater intensity than before. Physical stress could be reduced by allowing regular rest breaks during the working day. The health risks associated with long and atypical working hours could be reduced by adhering to daily maximum working hours and requiring recovery phases between shifts in critical jobs. Such a balance of service provision and staff safety is all the more necessary to prevent burnout and insomnia under the increased workloads caused by the COVID-19 pandemic. To maintain the working capacity of this highly strained group of employees, the work-related disadvantages and strains of close physical proximity to others, heavy physical demands and inconvenient working time patterns need to be addressed as a whole through different measures, as physical exhaustion often leads to individual failures to comply with occupational health and safety measures.

## Supplementary Information

**Additional file 1.** **Appendix:** **Table A1**: Critical occupations according to Burstedde et al. (2008: 27ff.). **Table A2**: Descriptive statistics for explanatory variables. **Table A3**: Robustness checks of the determinants of working in a critical job (logistic regressions). **Table A4**: Robustness checks for working conditions in critical jobs.

## Data Availability

The Scientific Use File of the BAuA Working Time Survey 2019, Version 1 (1048697/baua.azb19.suf.1) can be obtained from the BAuA research data centre (https://www.baua.de/DE/Angebote/Forschungsdaten/Arbeitszeitbefragung.html).

## Footnotes

1 The first-hour critical occupations consist of those corresponding to activities considered systemically relevant since the beginning of the coronavirus crisis.

2 The German classification of occupations (KldB 2010) comprises five digits: the first four digits categorise occupations on a horizontal dimension according to the area of expertise; the fifth digit groups occupations on a vertical dimension according to four requirement levels and, thus, the complexity of the tasks performed. The first digit level consists of 10 occupational areas, the second of 37 occupational main groups, the third of 144 occupational groups, the fourth of 700 occupational subgroups, and the fifth of 1,286 occupational types. Another aggregation level covers 14 occupational segments, which are used in the following regression analyses in Table 3 to characterise critical occupations.

3 There are currently almost 28,000 different occupational titles in Germany. In addition to the common, current occupational and task designations, these also include synonyms and related forms of these designations, precursor occupations, former GDR occupations and other common designations relevant to the labour market. These individual positions are kept by the Federal Employment Agency (BA) in an occupational database and are clearly assigned to an occupational type of the KldB (2010) classification.

4 The value 4.33 denotes the average number of weeks per month.

5 Descriptive statistics on the explanatory variables are provided in the appendix (Additional file 1: Appendix Table A2).

6 The complete regression results for models 4 to 9 can be obtained from the authors upon request.

7 Since the regression coefficients indicate log points, these can be transformed into percentages by using the formula $\left({e}^{\beta }-1\right)*100$.

8 The alternative three-digit level classification of first-hour occupations by Koebe et al. (2020) does not especially contain occupations in farming, animal husbandry, fishing, underground and surface mining, plastic- and rubber-making and -processing, paper-processing and packaging, precision mechanics, machine-building and -operating, the automotive, aeronautic, aerospace and ship building industries, energy technologies, electrical engineering, the production of clothing and other textile products, the production of foodstuffs, water resource management, civil engineering, building services engineering, plumping, sanitation, heating, ventilating, and air conditioning, biology, chemical and pharmaceutical engineering, environmental protection management, software development and programming, drivers and operators of construction and transportation vehicles and equipment, occupations in medical documentation, teachers in schools of general education, editors and journalists, and technical occupations in video and sound production. In contrast to the classification by Burstedde et al. (2020), the categorization by Koebe et al. (2020) primarily additionally includes occupations in plant, vessels, tank and apparatus construction, sales occupations (retail trade) selling office supplies, gifts and toys, gastronomy occupations, occupations in recruiting and employment services, insurance and financial services, tax consultancy, non-medical animal health practitioners, and occupations in non-medical therapy and alternative medicine.

9 The KRITIS list does not especially include occupations in public administration, biology, chemistry, cooking occupations, doctors’ receptionists and assistants, drivers of vehicles in road traffic, occupations in editorial work and journalism, transport and logistics, occupations in legal services, jurisdiction, and other officers of the court, IT-network engineering, IT-coordination, IT-administration and IT-organisation, occupations in education and social work, and pedagogic specialists in social care work, nursing, emergency medical services and obstetrics, physical security, personal protection, fire protection and workplace safety, teachers in schools of general education, occupations in cleaning services, building services engineering, funeral services, geriatric care, electrical engineering, technical occupations in energy technologies, occupations in purchasing and sales, plumping, sanitation, heating, ventilating, and air conditioning. This list additionally contains sales occupations in retail trade, occupations in non-medical therapy and alternative medicine, gastronomy occupations, media, documentation and information services, advertising and marketing, business organisation and strategy, accounting, controlling and auditing, and purchasing and sales. Generally, the disadvantage of the KRITIS list categorization is that it excludes entire sectors of the economy with occupations that are critical or includes entire sectors of the economy even though they contain occupations that are not critical.

10 Since this classification is based on three-digit occupations, occupational segments could not be included in the estimation due to multicollinearity.
