# Supplementary material for: COVID-19 and the labour market: What are the working conditions in critical jobs?
Source: J Labour Mark Res. 2022 Jul 26;56(1):10. doi: 10.1186/s12651-022-00315-6 (PMC9321290; doi:10.1186/s12651-022-00315-6)
Supplement: Supplementary file 1 — Additional file 1. Appendix: Table A1: Critical occupations according to Burstedde et al. (2008: 27ff.). Table A2: Descriptive statistics for explanatory variables. Table A3: Robustness checks of the determinants of working in a critical job (logistic regressions). Table A4: Robustness checks for working conditions in critical jobs. [file 12651_2022_315_MOESM1_ESM.docx]

**Appendix**

Table A1: Critical occupations according to Burstedde et al. (2008: 27ff.)

| **Title of occupational types** | **Identifier** |
| --- | --- |
| Occupations in farming (without specialisation)-unskilled/semiskilled tasks | 11101 |
| Occupations in farming (without specialisation)-skilled tasks | 11102 |
| Occupations in farming (without specialisation)-complex tasks | 11103 |
| Occupations in farming (without specialisation)-highly complex tasks | 11104 |
| Technical occupations in farming-complex tasks | 11113 |
| Technical occupations in farming-highly complex tasks | 11114 |
| Occupations in farming (with specialisation, not elsewhere classified)-skilled tasks | 11182 |
| Occupations in farming (with specialisation, not elsewhere classified)-complex tasks | 11183 |
| Occupations in farming (with specialisation, not elsewhere classified)-highly complex tasks | 11184 |
| Supervisors in farming | 11193 |
| Managers in farming | 11194 |
| Occupations in livestock farming (without poultry farming)-unskilled/semiskilled tasks | 11211 |
| Occupations in livestock farming (without poultry farming)-skilled tasks | 11212 |
| Occupations in livestock farming (without poultry farming)-complex tasks | 11213 |
| Occupations in livestock farming (without poultry farming)-highly complex tasks | 11214 |
| Occupations in poultry farming-skilled tasks | 11222 |
| Occupations in poultry farming-complex tasks | 11223 |
| Occupations in animal husbandry (with specialisation, not elsewhere classified)-skilled tasks | 11282 |
| Occupations in animal husbandry (with specialisation, not elsewhere classified)-complex tasks | 11283 |
| Supervisors in animal husbandry | 11293 |
| Managers in animal husbandry | 11294 |
| Occupations in fishing (without specialisation)-unskilled/semiskilled tasks | 11401 |
| Occupations in fishing (without specialisation)-skilled tasks | 11402 |
| Occupations in fish farming-skilled tasks | 11412 |
| Occupations in fishery-skilled tasks | 11422 |
| Occupations in fishery-complex tasks | 11423 |
| Occupations in fishery-highly complex tasks | 11424 |
| Supervisors in fishing | 11493 |
| Managers in fishing | 11494 |
| Occupations in livestock care-skilled tasks | 11512 |
| Occupations in fruit and vegetable farming-skilled tasks | 12112 |
| Occupations in fruit and vegetable farming-complex tasks | 12113 |
| Occupations in underground and surface mining-unskilled/semiskilled tasks | 21111 |
| Occupations in underground and surface mining-skilled tasks | 21112 |
| Occupations in underground and surface mining-complex tasks | 21113 |
| Occupations in underground and surface mining-highly complex tasks | 21114 |
| Supervisors in underground and surface mining and blasting engineering | 21193 |
| Managers in underground and surface mining and blasting engineering | 21194 |
| Occupations in precision optics-skilled tasks | 21362 |
| Occupations in precision optics-complex tasks | 21363 |
| Occupations in plastic- and rubber-making (without specialisation)-unskilled/semiskilled tasks | 22101 |
| Occupations in plastic- and rubber-making (without specialisation)-skilled tasks | 22102 |
| Occupations in plastic- and rubber-making (without specialisation)-complex tasks | 22103 |
| Occupations in plastic- and rubber-making (without specialisation)-highly complex tasks | 22104 |
| Technical occupations in tire production and vulcanisation-skilled tasks | 22112 |
| Occupations in plastic- and rubber-making and -processing (with specialisation, not elsewhere classified)-skilled tasks | 22182 |
| Occupations in plastic- and rubber-making and -processing (with specialisation, not elsewhere classified)-complex tasks | 22183 |
| Occupations in plastic- and rubber-making and -processing (with specialisation, not elsewhere classified)-highly complex tasks | 22184 |
| Supervisors in plastic- and rubber-making and -processing | 22193 |
| Technical occupations in paper-making and -processing and packaging (without specialisation)-unskilled/semiskilled tasks | 23101 |
| Occupations in paper-making (without specialisation)-skilled tasks | 23112 |
| Occupations in paper-making (without specialisation)-complex tasks | 23113 |
| Occupations in paper-making (without specialisation)-highly complex tasks | 23114 |
| Occupations in paper-processing and packaging-skilled tasks | 23122 |
| Occupations in paper-processing and packaging-complex tasks | 23123 |
| Occupations in paper-processing and packaging-highly complex tasks | 23124 |
| Supervisors in paper-making and -processing and packaging | 23193 |
| Occupations in precision mechanics-skilled tasks | 24512 |
| Occupations in precision mechanics-complex tasks | 24513 |
| Occupations in precision mechanics-highly complex tasks | 24514 |
| Supervisors in precision mechanics and tool making | 24593 |
| Technical service staff in maintenance and repair-skilled tasks | 25132 |
| Technical service staff in maintenance and repair-complex tasks | 25133 |
| Technical service staff in maintenance and repair-highly complex tasks | 25134 |
| Occupations in machine-building and -operating (with specialisation, not elsewhere classified)-skilled tasks | 25182 |
| Occupations in machine-building and -operating (with specialisation, not elsewhere classified)-complex tasks | 25183 |
| Supervisors in machine-building and -operating | 25193 |
| Managers in machine-building and -operating | 25194 |
| Technical occupations, agricultural and construction machinery-skilled tasks | 25222 |
| Technical occupations, agricultural and construction machinery-complex tasks | 25223 |
| Technical occupations, agricultural and construction machinery-highly complex tasks | 25224 |
| Technical occupations in the aeronautic and aerospace industries-skilled tasks | 25232 |
| Technical occupations in the aeronautic and aerospace industries-complex tasks | 25233 |
| Technical occupations in the aeronautic and aerospace industries-highly complex tasks | 25234 |
| Technical occupations in ship building-skilled tasks | 25242 |
| Technical occupations in ship building-complex tasks | 25243 |
| Technical occupations in ship building-highly complex tasks | 25244 |
| Supervisors in the automotive, aeronautic, aerospace and ship building industries | 25293 |
| Managers in the automotive, aeronautic, aerospace and ship building industries | 25294 |
| Technical occupations in maintenance of electric machines-complex tasks | 26223 |
| Technical occupations in energy and power plant technology-skilled tasks | 26232 |
| Technical occupations in energy and power plant technology-complex tasks | 26233 |
| Technical occupations in energy and power plant technology-highly complex tasks | 26234 |
| Occupations in renewable energy technology-skilled tasks | 26242 |
| Occupations in renewable energy technology-complex tasks | 26243 |
| Occupations in renewable energy technology-highly complex tasks | 26244 |
| Occupations in installing and maintaining electrical machines and equipment in plants-skilled tasks | 26252 |
| Occupations in installing and maintaining electrical machines and equipment in plants-complex tasks | 26253 |
| Occupations in installing and servicing electrical cables-skilled tasks | 26262 |
| Occupations in installing and servicing electrical cables-complex tasks | 26263 |
| Occupations in installing and servicing electrical cables-highly complex tasks | 26264 |
| Supervisors in energy technology | 26293 |
| Occupations in information and telecommunication technology-skilled tasks | 26312 |
| Occupations in information and telecommunication technology-complex tasks | 26313 |
| Occupations in information and telecommunication technology-highly complex tasks | 26314 |
| Occupations in aeronautic, naval, and automotive electronics-skilled tasks | 26332 |
| Occupations in in aeronautic, naval, and automotive electronics-complex tasks | 26333 |
| Occupations in in aeronautic, naval, and automotive electronics-highly complex tasks | 26334 |
| Occupations in electrical engineering (with specialisation, not elsewhere classified)-skilled tasks | 26382 |
| Occupations in electrical engineering (with specialisation, not elsewhere classified)-complex tasks | 26383 |
| Supervisors in electrical engineering | 26393 |
| Draftspersons-skilled tasks | 27212 |
| Occupations in technical design and apparatus building-complex tasks | 27223 |
| Occupations in technical design and apparatus building-highly complex tasks | 27224 |
| Occupations in textile production-skilled tasks | 28122 |
| Occupations in textile production-complex tasks | 28123 |
| Occupations in the production of clothing, hat and cap making-skilled tasks | 28222 |
| Occupations in the production of clothing, hat and cap making-complex tasks | 28223 |
| Supervisors in the production of clothing and other textile products | 28293 |
| Managers in the production of clothing and other textile products | 28294 |
| Occupations in the production of foodstuffs (without specialisation)-unskilled/semiskilled tasks | 29201 |
| Occupations in the production of foodstuffs (without specialisation)-skilled tasks | 29202 |
| Occupations in the production of foodstuffs (without specialisation)-complex tasks | 29203 |
| Occupations in the production of foodstuffs (without specialisation)-highly complex tasks | 29204 |
| Occupations in the production of milling products and animal feeds-skilled tasks | 29212 |
| Occupations in the production of milling products and animal feeds-complex tasks | 29213 |
| Occupations in the production of baked goods and pastries-skilled tasks | 29222 |
| Occupations in the production of baked goods and pastries-complex tasks | 29223 |
| Occupations in meat processing-skilled tasks | 29232 |
| Occupations in meat processing-complex tasks | 29233 |
| Occupations in fish processing-skilled tasks | 29242 |
| Occupations in fish processing-complex tasks | 29243 |
| Occupations in the production of dairy goods-skilled tasks | 29252 |
| Occupations in the production of dairy goods-complex tasks | 29253 |
| Occupations in the production of foodstuffs (with specialisation, not elsewhere classified)-skilled tasks | 29282 |
| Occupations in the production of foodstuffs (with specialisation, not elsewhere classified)-complex tasks | 29283 |
| Occupations in the production of foodstuffs (with specialisation, not elsewhere classified)-highly complex tasks | 29284 |
| Supervisors in the production of foodstuffs, confectionery and tobacco products | 29293 |
| Managers in the production of foodstuffs, confectionery and tobacco products | 29294 |
| Cooks (without specialisation)-unskilled/semiskilled tasks | 29301 |
| Cooks (without specialisation)-skilled tasks | 29302 |
| Supervisors in cooking | 29393 |
| Managers in cooking | 29394 |
| Occupations in water resource management-skilled tasks | 31142 |
| Occupations in water resource management-complex tasks | 31143 |
| Occupations in water resource management-highly complex tasks | 31144 |
| Occupations in road and asphalt construction-skilled tasks | 32222 |
| Occupations in road and asphalt construction-complex tasks | 32223 |
| Occupations in road and asphalt construction-highly complex tasks | 32224 |
| Occupations in railroad construction-skilled tasks | 32232 |
| Occupations in railroad construction-complex tasks | 32233 |
| Occupations in canal and tunnel construction-skilled tasks | 32252 |
| Occupations in canal and tunnel construction-complex tasks | 32253 |
| Occupations in land improvement and hydraulic construction-skilled tasks | 32262 |
| Occupations in land improvement and hydraulic construction-complex tasks | 32263 |
| Occupations in land improvement and hydraulic construction-highly complex tasks | 32264 |
| Supervisors in civil engineering | 32293 |
| Occupations in building services engineering (without specialisation)-skilled tasks | 34102 |
| Occupations in building services engineering (without specialisation)-complex tasks | 34103 |
| Occupations in building services engineering (without specialisation)-highly complex tasks | 34104 |
| Supervisors in building services engineering | 34193 |
| Occupations in sanitation, heating, ventilating, and air conditioning-skilled tasks | 34212 |
| Occupations in sanitation, heating, ventilating, and air conditioning-complex tasks | 34213 |
| Occupations in ventilating, and air conditioning-skilled tasks | 34232 |
| Occupations in ventilating, and air conditioning-complex tasks | 34233 |
| Supervisors in sanitation, heating, ventilating, and air conditioning | 34293 |
| Occupations in building services and waste disposal (without specialisation)-unskilled/semiskilled tasks | 34301 |
| Occupations in building services and waste disposal (without specialisation)-skilled tasks | 34302 |
| Occupations in building services and waste disposal (without specialisation)-complex tasks | 34303 |
| Technical occupations in water supply and wastewater disposal-skilled tasks | 34312 |
| Technical occupations in water supply and wastewater disposal-complex tasks | 34313 |
| Technical occupations in water supply and wastewater disposal -highly complex tasks | 34314 |
| Occupations in pipeline construction-skilled tasks | 34322 |
| Occupations in pipeline construction-complex tasks | 34323 |
| Occupations in waste management-skilled tasks | 34332 |
| Occupations in waste management-complex tasks | 34333 |
| Occupations in waste management-highly complex tasks | 34334 |
| Supervisors in building services and waste disposal | 34393 |
| Biological technical laboratory occupations-skilled tasks | 41212 |
| Biological technical laboratory occupations-complex tasks | 41213 |
| Biological technical laboratory occupations-highly complex tasks | 41214 |
| Occupations in the preparation of biological specimen-skilled tasks | 41222 |
| Occupations in microbiology-highly complex tasks | 41264 |
| Occupations in human biology-highly complex tasks | 41274 |
| Occupations in biology (with specialisation, not elsewhere classified)-complex tasks | 41283 |
| Occupations in biology (with specialisation, not elsewhere classified)-highly complex tasks | 41284 |
| Supervisors in biology | 41293 |
| Managers in biology | 41294 |
| Occupations in chemical and pharmaceutical engineering-unskilled/semiskilled tasks | 41311 |
| Occupations in chemical and pharmaceutical engineering-skilled tasks | 41312 |
| Occupations in chemical and pharmaceutical engineering-complex tasks | 41313 |
| Occupations in chemical and pharmaceutical engineering-highly complex tasks | 41314 |
| Chemical technical laboratory occupations-skilled tasks | 41322 |
| Chemical technical laboratory occupations-complex tasks | 41323 |
| Chemical technical laboratory occupations-highly complex tasks | 41324 |
| Operators of chemical production plants-complex tasks | 41333 |
| Operators of oil and gas refinery plants-complex tasks | 41343 |
| Occupations in chemistry (with specialisation, not elsewhere classified)-complex tasks | 41383 |
| Occupations in chemistry (with specialisation, not elsewhere classified)-highly complex tasks | 41384 |
| Supervisors in chemistry | 41393 |
| Managers in chemistry | 41394 |
| Water pollution and emission control, waste management commissioners-complex tasks | 42323 |
| Water pollution and emission control, waste management commissioners-highly complex tasks | 42324 |
| Radiation protection commissioners-complex tasks | 42333 |
| Radiation protection commissioners-highly complex tasks | 42334 |
| Managers in environmental protection administration and consulting | 42394 |
| Occupations in computer science (without specialisation)-skilled tasks | 43102 |
| Occupations in computer science (without specialisation)-complex tasks | 43103 |
| Occupations in computer science (without specialisation)-highly complex tasks | 43104 |
| Occupations in bio- and medical informatics-highly complex tasks | 43134 |
| Managers in computer science | 43194 |
| Occupations in IT-application-consulting-complex tasks | 43223 |
| Managers in IT-system-analysis, IT-application-consulting and IT-sales | 43294 |
| Occupations in IT-network engineering-complex tasks | 43313 |
| Occupations in IT-network engineering-highly complex tasks | 43314 |
| Occupations in IT-system-administration-complex tasks | 43343 |
| Occupations in web administration-complex tasks | 43363 |
| Occupations in IT-network engineering, IT-coordination, IT-administration and IT-organisation (with specialisation, not elsewhere classified)-complex tasks | 43383 |
| Occupations in IT-network engineering, IT-coordination, IT-administration and IT-organisation (with specialisation, not elsewhere classified)-highly complex tasks | 43384 |
| Managers in IT-network engineering, IT-coordination, IT-administration and IT-organisation (with specialisation, not elsewhere classified) | 43394 |
| Occupations in software development-skilled tasks | 43412 |
| Occupations in software development-complex tasks | 43413 |
| Occupations in software development-highly complex tasks | 43414 |
| Occupations in programming-complex tasks | 43423 |
| Managers in software development and programming | 43494 |
| Technical occupations in railway operation-skilled tasks | 51112 |
| Technical occupations in railway operation-complex tasks | 51113 |
| Technical occupations in aircraft operation-skilled tasks | 51122 |
| Technical occupations in aircraft operation-complex tasks | 51123 |
| Technical occupations in ship operation-skilled tasks | 51132 |
| Technical occupations in ship operation-complex tasks | 51133 |
| Technical occupations in ship operation-highly complex tasks | 51134 |
| Technical occupations in railway, aircraft and ship operation (with specialisation, not elsewhere classified)-skilled tasks | 51182 |
| Technical occupations in railway, aircraft and ship operation (with specialisation, not elsewhere classified)-complex tasks | 51183 |
| Supervisors in railway, aircraft and ship operation | 51193 |
| Road and tunnel inspection and controlling commissioners-skilled tasks | 51212 |
| Occupations in the inspection and maintenance of railway infrastructure-skilled tasks | 51222 |
| Occupations in the inspection and maintenance of railway infrastructure-complex tasks | 51223 |
| Occupations in the inspection and maintenance of railway infrastructure-highly complex tasks | 51224 |
| Technical occupations in air traffic control-complex tasks | 51233 |
| Technical occupations in air traffic control-highly complex tasks | 51234 |
| Waterway and bridges inspection and controlling commissioners-skilled tasks | 51242 |
| Waterway and bridges inspection and controlling commissioners-complex tasks | 51243 |
| Supervisors in the inspection and maintenance of traffic infrastructure | 51293 |
| Occupations in warehousing and logistics-unskilled/semiskilled tasks | 51311 |
| Occupations in warehousing and logistics-skilled tasks | 51312 |
| Occupations in postal and other delivery services-unskilled/semiskilled tasks | 51321 |
| Occupations in postal and other delivery services-skilled tasks | 51322 |
| Occupations in cargo handling-skilled tasks | 51332 |
| Supervisors in warehousing and logistics, in postal and other delivery services, and in cargo handling | 51393 |
| Managers in warehousing and logistics, in postal and other delivery services, and in cargo handling | 51394 |
| Service occupations in road and railway traffic-skilled tasks | 51412 |
| Service occupations in air traffic-skilled tasks | 51422 |
| Service occupations in shipping traffic-skilled tasks | 51432 |
| Supervisors of service personnel in passenger traffic | 51493 |
| Occupations in traffic surveillance and control (without specialisation)-complex tasks | 51503 |
| Occupations in traffic surveillance and control (without specialisation)-highly complex tasks | 51504 |
| Occupations in the surveillance and control of road traffic-skilled tasks | 51512 |
| Occupations in the surveillance and control of road traffic-complex tasks | 51513 |
| Occupations in the surveillance and control of railway traffic-skilled tasks | 51522 |
| Occupations in the surveillance and control of railway traffic-complex tasks | 51523 |
| Occupations in the surveillance and control of air traffic-skilled tasks | 51532 |
| Occupations in the surveillance and control of air traffic-complex tasks | 51533 |
| Occupations in the surveillance and control of air traffic-highly complex tasks | 51534 |
| Occupations in the surveillance and control of shipping traffic-complex tasks | 51543 |
| Occupations in traffic surveillance and control (with specialisation, not elsewhere classified)-complex tasks | 51583 |
| Supervisors in traffic surveillance and control | 51593 |
| Managers in traffic surveillance and control | 51594 |
| Management assistants in transport-complex tasks | 51613 |
| Management assistants in transport -highly complex tasks | 51614 |
| Forwarding agents and management assistants in logistics-skilled tasks | 51622 |
| Forwarding agents and management assistants in logistics-complex tasks | 51623 |
| Forwarding agents and management assistants in logistics-highly complex tasks | 51624 |
| Management assistants in road and railway transport-skilled tasks | 51632 |
| Management assistants in road and railway transport-complex tasks | 51633 |
| Management assistants in air transport-skilled tasks | 51642 |
| Management assistants in air transport-complex tasks | 51643 |
| Management assistants in shipping transport-skilled tasks | 51652 |
| Management assistants in shipping transport-complex tasks | 51653 |
| Management assistants in courier services, express and postal delivery services-skilled tasks | 51662 |
| Management assistants in courier services, express and postal delivery services-complex tasks | 51663 |
| Managers in transport and logistics | 51694 |
| Professional drivers (passengers transport)-skilled tasks | 52112 |
| Professional drivers (cargo trucks)-skilled tasks | 52122 |
| Bus and tram drivers-skilled tasks | 52132 |
| Drivers of vehicles in road traffic (with specialisation, not elsewhere classified)-skilled tasks | 52182 |
| Drivers of train engines and other railway vehicles-skilled tasks | 52202 |
| Pilots of planes and airliners-complex tasks | 52313 |
| Pilots of planes and airliners-highly complex tasks | 52314 |
| Aircraft pilots (with specialisation, not elsewhere classified)-complex tasks | 52383 |
| Aircraft pilots (with specialisation, not elsewhere classified)-highly complex tasks | 52384 |
| Deck officers/mates and ship’s captains or skippers-complex tasks | 52413 |
| Deck officers/mates and ship’s captains or skippers-highly complex tasks | 52414 |
| Ship’s masters in inland navigation and port traffic-skilled tasks | 52422 |
| Ship’s masters in inland navigation and port traffic-complex tasks | 52423 |
| Drivers of agricultural and forestry machines-skilled tasks | 52512 |
| Operators of cranes, lifts and related lifting devices-unskilled/semiskilled tasks | 52531 |
| Operators of cranes, lifts and related lifting devices-skilled tasks | 52532 |
| Supervisors of drivers and operators of construction and transportation vehicles and equipment | 52593 |
| Occupations in physical security, protection of valuables, and personal protection-unskilled/semiskilled tasks | 53111 |
| Occupations in physical security, protection of valuables, and personal protection-skilled tasks | 53112 |
| Occupations focusing on workplace safety and safety technology-skilled tasks | 53122 |
| Occupations focusing on workplace safety and safety technology-complex tasks | 53123 |
| Occupations focusing on workplace safety and safety technology-highly complex tasks | 53124 |
| Occupations in fire protection-skilled tasks | 53132 |
| Occupations in fire protection-complex tasks | 53133 |
| Occupations in fire protection-highly complex tasks | 53134 |
| Supervisors in physical security, personal protection, fire protection and workplace safety | 53193 |
| Managers in physical security, personal protection, fire protection and workplace safety | 53194 |
| Uniformed police personnel-skilled tasks | 53212 |
| Uniformed police personnel-complex tasks | 53213 |
| Uniformed police personnel-highly complex tasks | 53214 |
| Detectives and police officers in criminal investigation departments-skilled tasks | 53222 |
| Detectives and police officers in criminal investigation departments-complex tasks | 53223 |
| Detectives and police officers in criminal investigation departments-highly complex tasks | 53224 |
| Police officers in penal institutions- unskilled/semiskilled tasks | 53241 |
| Police officers in penal institutions-skilled tasks | 53242 |
| Police officers in penal institutions-complex tasks | 53243 |
| Police officers in penal institutions-highly complex tasks | 53244 |
| Occupations in public health authority and hygiene control-skilled tasks | 53322 |
| Occupations in public health authority and hygiene control-complex tasks | 53323 |
| Occupations in food control-skilled tasks | 53332 |
| Occupations in food control-complex tasks | 53333 |
| Occupations in disinfection and pest control-skilled tasks | 53342 |
| Supervisors in occupational health and safety administration, public health authority, and disinfection | 53393 |
| Managers in occupational health and safety administration, public health authority, and disinfection | 53394 |
| Occupations in cleaning services (without specialisation)-unskilled/semiskilled tasks | 54101 |
| Occupations in building cleaning services-skilled tasks | 54112 |
| Occupations in building cleaning services-complex tasks | 54113 |
| Occupations in textile cleaning services-skilled tasks | 54132 |
| Occupations in machine and equipment cleaning services-skilled tasks | 54142 |
| Supervisors in cleaning services | 54193 |
| Occupations in purchasing-skilled tasks | 61112 |
| Occupations in purchasing-complex tasks | 61113 |
| Managers in purchasing and sales | 61194 |
| Management assistants in trade (without specialisation)-complex tasks | 61203 |
| Management assistants in trade (without specialisation)-highly complex tasks | 61204 |
| Management assistants in wholesale and foreign trade-skilled tasks | 61212 |
| Management assistants in wholesale and foreign trade-complex tasks | 61213 |
| Management assistants in wholesale and foreign trade-highly complex tasks | 61214 |
| Managers in trade | 61294 |
| Cashiers and ticket agents-skilled tasks | 62112 |
| Stall and market sellers-skilled tasks | 62122 |
| Supervisors in retail trade | 62193 |
| Managers in retail trade | 62194 |
| Sales occupations (retail trade) in gardening stores, home improvement stores, pet shops and zoo supply stores-skilled tasks | 62262 |
| Sales occupations (retail) selling foodstuffs (without specialisation)-unskilled/semiskilled tasks | 62301 |
| Sales occupations (retail) selling foodstuffs (without specialisation)-skilled tasks | 62302 |
| Sales occupations (retail) selling baked goods, pastries and confectionaries-skilled tasks | 62312 |
| Sales occupations (retail) selling meat products-skilled tasks | 62322 |
| Sales occupations (retail) selling foodstuffs (with specialisation, not elsewhere classified)-skilled tasks | 62382 |
| Sales occupations (retail) selling drugstore products and pharmaceuticals-skilled tasks | 62412 |
| Sales occupations (retail) selling medical supplies and healthcare goods-skilled tasks | 62422 |
| Legislators-highly complex tasks | 71214 |
| Occupations in human resources development and personnel service-skilled tasks | 71512 |
| Occupations in human resources development and personnel service-complex tasks | 71513 |
| Occupations in human resources development and personnel service-highly complex tasks | 71514 |
| Managers in human resources management and personnel services | 71594 |
| Bankers-skilled tasks | 72112 |
| Bankers-complex tasks | 72113 |
| Insurance salespersons-skilled tasks | 72132 |
| Insurance salespersons-complex tasks | 72133 |
| Insurance salespersons-highly complex tasks | 72134 |
| Managers in insurance and financial services | 72194 |
| Notaries-highly complex tasks | 73124 |
| Lawyers-highly complex tasks | 73134 |
| Prosecutors-highly complex tasks | 73144 |
| Judges-highly complex tasks | 73154 |
| Occupations in the national security service-skilled tasks | 73162 |
| Occupations in the national security service-complex tasks | 73163 |
| Occupations in the national security service-highly complex tasks | 73164 |
| Managers in legal services, jurisdiction, and of other officers of the court | 73194 |
| Occupations in public administration (without specialisation)-unskilled/semiskilled tasks | 73201 |
| Occupations in public administration (without specialisation)-skilled tasks | 73202 |
| Occupations in public administration (without specialisation)-complex tasks | 73203 |
| Occupations in public administration (without specialisation)-highly complex tasks | 73204 |
| Occupations in the social service administration and the social security system-skilled tasks | 73212 |
| Occupations in the social service administration and the social security system-complex tasks | 73213 |
| Occupations in the social service administration and the social security system-highly complex tasks | 73214 |
| Administrative occupations in the welfare and health care system-skilled tasks | 73222 |
| Administrative occupations in the welfare and health care system-complex tasks | 73223 |
| Administrative occupations in the welfare and health care system-highly complex tasks | 73224 |
| Occupations in tax administration-unskilled/semiskilled tasks | 73231 |
| Occupations in tax administration-skilled tasks | 73232 |
| Occupations in tax administration-complex tasks | 73233 |
| Occupations in tax administration-highly complex tasks | 73234 |
| Occupations in the customs service-unskilled/semiskilled tasks | 73241 |
| Occupations in the customs service-skilled tasks | 73242 |
| Occupations in the customs service-complex tasks | 73243 |
| Occupations in the customs service-highly complex tasks | 73244 |
| Occupations in the administration of justice-skilled tasks | 73252 |
| Occupations in the administration of justice-complex tasks | 73253 |
| Occupations in the administration of justice-highly complex tasks | 73254 |
| Occupations in public administration (with specialisation, not elsewhere classified)-skilled tasks | 73282 |
| Occupations in public administration (with specialisation, not elsewhere classified)-complex tasks | 73283 |
| Occupations in public administration (with specialisation, not elsewhere classified)-highly complex tasks | 73284 |
| Supervisors in public administration | 73293 |
| Managers in public administration | 73294 |
| Occupations in medical documentation-skilled tasks | 73342 |
| Managers in media, documentation and information services | 73394 |
| Medical assistants (without specialisation)-skilled tasks | 81102 |
| Medical assistants (without specialisation)-complex tasks | 81103 |
| Dental assistants-skilled tasks | 81112 |
| Dental assistants-complex tasks | 81113 |
| Orthoptists-skilled tasks | 81132 |
| Veterinary assistants-skilled tasks | 81142 |
| Veterinary assistants-complex tasks | 81143 |
| Medical assistants (with specialisation, not elsewhere classified)-skilled tasks | 81182 |
| Medical assistants (with specialisation, not elsewhere classified)-complex tasks | 81183 |
| Technical laboratory occupations in medicine-skilled tasks | 81212 |
| Technical laboratory occupations in medicine-complex tasks | 81213 |
| Technical laboratory occupations in medicine-highly complex tasks | 81214 |
| Technical occupations in medical laboratories for functional diagnostics-skilled tasks | 81222 |
| Technical occupations in medical laboratories for functional diagnostics-complex tasks | 81223 |
| Technical occupations in medical laboratories for functional diagnostics-highly complex tasks | 81224 |
| Technical occupations in radiology-skilled tasks | 81232 |
| Technical occupations in radiology-complex tasks | 81233 |
| Technical occupations in radiology-highly complex tasks | 81234 |
| Technical occupations in veterinary medicine-skilled tasks | 81242 |
| Technical occupations in veterinary medicine-complex tasks | 81243 |
| Managers in medical laboratories | 81294 |
| Occupations in nursing (without specialisation)-unskilled/semiskilled tasks | 81301 |
| Occupations in nursing (without specialisation)-skilled tasks | 81302 |
| Occupations in nursing specialised in a particular branch of nursing-complex tasks | 81313 |
| Occupations in nursing specialised in paediatrics-complex tasks | 81323 |
| Surgical and medico-technical assistants-skilled tasks | 81332 |
| Surgical and medico-technical assistants-complex tasks | 81333 |
| Occupations in emergency medical services-unskilled/semiskilled tasks | 81341 |
| Occupations in emergency medical services-skilled tasks | 81342 |
| Occupations in emergency medical services-complex tasks | 81343 |
| Occupations in obstetrics and maternity care-skilled tasks | 81352 |
| Occupations in obstetrics and maternity care-complex tasks | 81353 |
| Occupations in nursing (with specialisation, not elsewhere classified)-skilled tasks | 81382 |
| Occupations nursing (with specialisation, not elsewhere classified)-complex tasks | 81383 |
| Supervisors in nursing, emergency medical services and obstetrics | 81393 |
| Managers in nursing, emergency medical services and obstetrics | 81394 |
| Medical doctors (without specialisation)-highly complex tasks | 81404 |
| Medical doctors specialised in pediatrics and adolescent medicine-highly complex tasks | 81414 |
| Medical doctors specialised in internal medicine-highly complex tasks | 81424 |
| Medical doctors specialised in surgery-highly complex tasks | 81434 |
| Medical doctors specialised in dermatology, otorhinolaryngology, ophthalmology, gynaecology, andrology and related medical fields-highly complex tasks | 81444 |
| Medical doctors specialised in anaesthesiology-highly complex tasks | 81454 |
| Medical doctors specialised in neurology, psychiatry, psychotherapy and psychosomatic medicine-highly complex tasks | 81464 |
| Dentists and orthodontists-highly complex tasks | 81474 |
| Medical doctors (with specialisation, not elsewhere classified)-highly complex tasks | 81484 |
| Managers in human medicine and dentistry | 81494 |
| Veterinaries (without specialisation)-highly complex tasks | 81504 |
| Veterinaries for large and farm animals-highly complex tasks | 81514 |
| Veterinaries for pets-highly complex tasks | 81524 |
| Managers in veterinary medicine and non-medical animal health practitioners | 81594 |
| Occupations in non-clinical psychology-highly complex tasks | 81614 |
| Occupations in clinical psychology-complex tasks | 81623 |
| Occupations in clinical psychology-highly complex tasks | 81624 |
| Occupations in non-medical psychotherapy-highly complex tasks | 81634 |
| Occupations in physiotherapy-skilled tasks | 81712 |
| Occupations in physiotherapy-complex tasks | 81713 |
| Occupations in physiotherapy-highly complex tasks | 81714 |
| Managers in in non-medical therapy and alternative medicine | 81794 |
| Pharmacists-highly complex tasks | 81804 |
| Medical doctors specialised in pharmacology-highly complex tasks | 81814 |
| Pharmaceutical-technical assistants-skilled tasks | 81822 |
| Occupations in pharmacy (with specialisation, not elsewhere classified)-complex tasks | 81883 |
| Occupations in pharmacy (with specialisation, not elsewhere classified)-highly complex tasks | 81884 |
| Managers in pharmacy | 81894 |
| Occupations in geriatric care (without specialisation)-unskilled/semiskilled tasks | 82101 |
| Occupations in geriatric care (without specialisation)-skilled tasks | 82102 |
| Occupations in geriatric care (without specialisation)-complex tasks | 82103 |
| Occupations in geriatric care (with specialisation, not elsewhere classified)-skilled tasks | 82182 |
| Occupations in geriatric care (with specialisation, not elsewhere classified)-complex tasks | 82183 |
| Managers in geriatric care | 82194 |
| Occupations in funeral services-skilled tasks | 82402 |
| Occupations in funeral services-complex tasks | 82403 |
| Supervisors in funeral services | 82493 |
| Managers in funeral services | 82494 |
| Technical occupations in medicine (without specialisation)-skilled tasks | 82502 |
| Technical occupations in medicine (without specialisation)-complex tasks | 82503 |
| Technical occupations in medicine (without specialisation)-highly complex tasks | 82504 |
| Technical occupations in orthopaedic and rehabilitation-skilled tasks | 82512 |
| Technical occupations in orthopaedic and rehabilitation-complex tasks | 82513 |
| Technical occupations in orthopaedic and rehabilitation-highly complex tasks | 82514 |
| Occupations in ophthalmic optics-skilled tasks | 82522 |
| Occupations in ophthalmic optics-complex tasks | 82523 |
| Occupations in ophthalmic optics-highly complex tasks | 82524 |
| Occupations in hearing-aid acoustics-skilled tasks | 82532 |
| Occupations in hearing-aid acoustics-complex tasks | 82533 |
| Occupations in hearing-aid acoustics-highly complex tasks | 82534 |
| Supervisors in medicine, orthopaedic and rehabilitation technology | 82593 |
| Managers in medicine, orthopaedic and rehabilitation technology | 82594 |
| Occupations in child care and child-rearing-unskilled/semiskilled tasks | 83111 |
| Occupations in child care and child-rearing-skilled tasks | 83112 |
| Occupations in social work and social pedagogics-complex tasks | 83123 |
| Occupations in social work and social pedagogics-highly complex tasks | 83124 |
| Pedagogic specialists in social care work and special needs education-unskilled/semiskilled tasks | 83131 |
| Pedagogic specialists in social care work and special needs education-skilled tasks | 83132 |
| Pedagogic specialists in social care work and special needs education-complex tasks | 83133 |
| Pedagogic specialists in social care work and special needs education-highly complex tasks | 83134 |
| Occupations in social, educational and addiction counselling-highly complex tasks | 83154 |
| Supervisors in education and social work, and of pedagogic specialists in social care work | 83193 |
| Managers in education and social work, and of pedagogic specialists in social care work | 83194 |
| Teachers in primary education-highly complex tasks | 84114 |
| Teachers in secondary education-highly complex tasks | 84124 |
| Teachers in schools for special needs education-highly complex tasks | 84134 |
| Managers in schools of general education | 84194 |
| Editors and journalists-skilled tasks | 92412 |
| Editors and journalists-complex tasks | 92413 |
| Editors and journalists-highly complex tasks | 92414 |
| Managers in editorial work and journalism | 92494 |
| Radio and television presenters-highly complex tasks | 94334 |
| Cinematographers, camera assistants, and projectionists-skilled tasks | 94522 |
| Technical occupations in video and sound production-skilled tasks | 94532 |
| Technical occupations in video and sound production-complex tasks | 94533 |
| Technical occupations in video and sound production-highly complex tasks | 94534 |
| Supervisors in event technology, cinematography, and sound engineering | 94593 |

Notes: The titles of the occupational types and the identifiers were obtained from the German classification of occupations (KldB 2010).

Table A2: Descriptive statistics for explanatory variables

|  | Mean | Standard deviation | Minimum | Maximum |
| --- | --- | --- | --- | --- |
| Gender (1 = female) | 0.47 | 0.499 | 0 | 1 |
| Age (in years) | 42.41 | 11.967 | 19 | 78 |
| Age squared (in years) | 1942.07 | 1030.629 | 361 | 6084 |
| Place of residence (1 = East Germany) | 0.19 | 0.389 | 0 | 1 |
| Highest professional degree |  |  |  |  |
| No professional degree | 0.04 | 0.196 | 0 | 1 |
| Vocational degree | 0.57 | 0.495 | 0 | 1 |
| Technical school, master | 0.13 | 0.341 | 0 | 1 |
| Polytechnic degree | 0.07 | 0.250 | 0 | 1 |
| University degree | 0.18 | 0.384 | 0 | 1 |
| Another degree | 0.01 | 0.098 | 0 | 1 |
| Unknown | 0.01 | 0.076 | 0 | 1 |
| Marital status |  |  |  |  |
| Single | 0.38 | 0.486 | 0 | 1 |
| Married | 0.50 | 0.500 | 0 | 1 |
| Civil union | 0.01 | 0.120 | 0 | 1 |
| Divorced/widowed | 0.10 | 0.306 | 0 | 1 |
| Unknown | 0.00 | 0.020 | 0 | 1 |
| Children in the household |  |  |  |  |
| No children in the household | 0.67 | 0.469 | 0 | 1 |
| Child younger than 7 years in the household | 0.13 | 0.338 | 0 | 1 |
| Child aged 7 to 12 years in the household | 0.11 | 0.317 | 0 | 1 |
| Child aged 13 to 18 years in the household | 0.08 | 0.275 | 0 | 1 |
| Tenure (in years) | 11.31 | 10.748 | 0 | 51 |
| Form of employment |  |  |  |  |
| Full-time | 0.75 | 0.435 | 0 | 1 |
| Part-time | 0.24 | 0.426 | 0 | 1 |
| Marginal employment | 0.01 | 0.121 | 0 | 1 |
| Unknown | 0.00 | 0.060 | 0 | 1 |
| Type of contract |  |  |  |  |
| Permanent contract | 0.90 | 0.294 | 0 | 1 |
| Fixed-term contract | 0.10 | 0.294 | 0 | 1 |
| Unknown | 0.05 | 0.222 | 0 | 1 |
| Complexity of job |  |  |  |  |
| Unskilled or semiskilled activity | 0.06 | 0.243 | 0 | 1 |
| Specialist activity | 0.57 | 0.495 | 0 | 1 |
| Complex specialist activity | 0.19 | 0.395 | 0 | 1 |
| Highly complex activity | 0.17 | 0.378 | 0 | 1 |
| Additional jobs |  |  |  |  |
| No additional job | 0.91 | 0.285 | 0 | 1 |
| One additional job | 0.08 | 0.273 | 0 | 1 |
| More than one additional job | 0.01 | 0.089 | 0 | 1 |
| Size of company |  |  |  |  |
| Fewer than 9 employees | 0.10 | 0.306 | 0 | 1 |
| 10-49 employees | 0.24 | 0.428 | 0 | 1 |
| 50-499 employees | 0.37 | 0.482 | 0 | 1 |
| More than 500 employees | 0.27 | 0.445 | 0 | 1 |
| Unknown | 0.02 | 0.127 | 0 | 1 |
| Work council |  |  |  |  |
| Existent | 0.59 | 0.492 | 0 | 1 |
| Nonexistent | 0.36 | 0.481 | 0 | 1 |
| Unknown | 0.05 | 0.215 | 0 | 1 |
| Occupational segments |  |  |  |  |
| Agriculture, forestry and gardening | 0.02 | 0.130 | 0 | 1 |
| Manufacturing | 0.07 | 0.251 | 0 | 1 |
| Manufacturing engineering | 0.13 | 0.340 | 0 | 1 |
| Construction | 0.07 | 0.252 | 0 | 1 |
| Food and hospitality | 0.04 | 0.199 | 0 | 1 |
| Medical and nonmedical health care | 0.11 | 0.316 | 0 | 1 |
| Social and cultural services | 0.11 | 0.307 | 0 | 1 |
| Retail and trade | 0.08 | 0.267 | 0 | 1 |
| Corporate management and organisation | 0.08 | 0.277 | 0 | 1 |
| Business services | 0.12 | 0.327 | 0 | 1 |
| IT and natural science services | 0.05 | 0.211 | 0 | 1 |
| Security | 0.03 | 0.160 | 0 | 1 |
| Transport and logistics | 0.09 | 0.281 | 0 | 1 |
| Cleaning | 0.01 | 0.109 | 0 | 1 |
| Number of observations | 7268 |  |  |  |

Notes: Results are weighted.

Source: Working Time Survey 2019; own calculations.

Table A3: Robustness checks of the determinants of working in a critical job (logistic regressions)

|  | Critical job— | Critical job— | Critical job— |
| --- | --- | --- | --- |
|  | Specification from model 3 of table 3 | Three-digit-level classification of Koebe et al. (2020) | Economic sectors of the original KRITIS list (Pfeiffer 2020) |
|  | (AME) | (AME) | (AME) |
| Gender (1 = female) | 0.080 | 0.127*** | 0.033 |
|  | (0.072) | (0.013) | (0.021) |
| Age (in years) | -0.006 | -0.009* | 0.000 |
|  | (0.025) | (0.005) | (0.005) |
| Age squared (in years) | -0.000 | 0.000* | -0.000 |
|  | (0.000) | (0.000) | (0.000) |
| Place of residence (1 = East Germany) | 0.079^*^ | 0.001 | -0.000^*^ |
|  | (0.032) | (0.006) | (0.005) |
| Highest professional degree (Ref.: University degree) |  |  |  |
| Vocational degree | -0.180 | 0.026 | -0.044^*^ |
|  | (0.100) | (0.019) | (0.019) |
| Technical school, master | -0.020 | 0.051* | -0.052^**^ |
|  | (0.111) | (0.021) | (0.019) |
| Polytechnic degree | 0.185 | 0.146*** | 0.010 |
|  | (0.098) | (0.020) | (0.039) |
| Another degree | 0.402 | 0.118* | -0.048 |
|  | (0.300) | (0.052) | (0.046) |
| No professional degree | -0.551^*^ | -0.012 | -0.030 |
|  | (0.243) | (0.041) | (0.035) |
| Unknown | -0.158 | 0.309** | -0.097 |
|  | (0.509) | (0.107) | (0.075) |
| Marital status (Ref.: Single) |  |  |  |
| Married | 0.402 | -0.003 | 0.025^*^ |
|  | (0.300) | (0.016) | (0.011) |
| Civil union | -0.551^*^ | 0.081 | -0.051 |
|  | (0.243) | (0.056) | (0.059) |
| Divorced/widowed | -0.158 | 0.037 | 0.011 |
|  | (0.509) | (0.020) | (0.016) |
| Unknown | 0.402 | 0.138 | -0.115 |
|  | (0.300) | (0.136) | (0.113) |
| Children in the household (Ref: No children in the household) |  |  |  |
| Child younger than 7 years in the household | -0.055 | -0.001 | -0.042^**^ |
|  | (0.111) | (0.022) | (0.015) |
| Child aged 7 to 12 years in the household | 0.085 | 0.033 | 0.006 |
|  | (0.105) | (0.020) | (0.014) |
| Child aged 13 to 18 years in the household | 0.140 | 0.019 | -0.035^*^ |
|  | (0.103) | (0.019) | (0.014) |
| Tenure (in years) | 0.009^**^ | 0.001 | 0.002^*^ |
|  | (0.003) | (0.001) | (0.001) |
| Form of employment (Ref.: Full-time) |  |  |  |
| Part-time | 0.107 | 0.100*** | -0.002 |
|  | (0.077) | (0.015) | (0.022) |
| Marginal employment | -0.716^*^ | 0.064 | 0.024 |
|  | (0.324) | (0.054) | (0.045) |
| Unknown | -2.200^**^ | -0.160 | -0.189 |
|  | (0.697) | (0.086) | (0.118) |
| Type of contract (1 = Permanent contract) |  |  |  |
| Fixed-term contract | -0.203 | -0.006 | 0.010 |
|  | (0.127) | (0.025) | (0.027) |
| Unknown | 1.063^***^ | 0.068*** | 0.180^**^ |
|  | (0.110) | (0.019) | (0.062) |
| Complexity of job (Ref.: Unskilled or semiskilled activity) |  |  |  |
| Specialist activity | -0.142 | 0.072* | 0.133^**^ |
|  | (0.189) | (0.032) | (0.047) |
| Complex specialist activity | -0.866^***^ | -0.210*** | -0.106 |
|  | (0.199) | (0.033) | (0.055) |
| Highly complex activity | -1.456^***^ | -0.288*** | 0.086 |
|  | (0.206) | (0.035) | (0.051) |
| Additional jobs (Ref.: No additional job) |  |  |  |
| One additional job | -0.255^*^ | 0.063** | 0.012 |
|  | (0.120) | (0.022) | (0.018) |
| More than one additional job | -0.417 | 0.054 | -0.004 |
|  | (0.312) | (0.057) | (0.041) |
| Size of company (Ref.: More than 500 employees) |  |  |  |
| Fewer than 9 employees | -0.089 | -0.005 | -0.041 |
|  | (0.130) | (0.024) | (0.047) |
| 10-49 employees | 0.204^*^ | 0.001 | -0.042 |
|  | (0.092) | (0.017) | (0.042) |
| 50-499 employees | 0.048 | -0.045** | -0.015 |
|  | (0.076) | (0.014) | (0.034) |
| Unknown | 0.107 | 0.053 | 0.026 |
|  | (0.276) | (0.051) | (0.053) |
| Work council (Ref.: Existent) |  |  |  |
| Nonexistent | -0.428^***^ | -0.094*** | -0.060 |
|  | (0.080) | (0.015) | (0.057) |
| Unknown | 0.067 | -0.031 | -0.009 |
|  | (0.196) | (0.034) | (0.071) |
| Occupational segments (Ref.: Manufacturing) |  |  |  |
| Agriculture, forestry and gardening | 0.670^*^ | -/- | 0.388^*^ |
|  | (0.301) | -/- | (0.193) |
| Manufacturing engineering | 1.219^***^ | -/- | 0.099^*^ |
|  | (0.177) | -/- | (0.046) |
| Construction | 1.295^***^ | -/- | 0.112 |
|  | (0.191) | -/- | (0.103) |
| Food and hospitality | 1.803^***^ | -/- | 0.313 |
|  | (0.227) | -/- | (0.167) |
| Medical and nonmedical health care | 4.273^***^ | -/- | 0.596^***^ |
|  | (0.218) | -/- | (0.146) |
| Social and cultural services | 2.398^***^ | -/- | -0.46 |
|  | (0.184) | -/- | (0.047) |
| Retail and trade | 0.700^***^ | -/- | 0.581^***^ |
|  | (0.191) | -/- | (0.127) |
| Corporate management and organisation | -0.580^**^ | -/- | 0.314^***^ |
|  | (0.206) | -/- | (0.077) |
| Business services | 1.775^***^ | -/- | 0.557^***^ |
|  | (0.176) | -/- | (0.101) |
| IT and natural science services | 3.000^***^ | -/- | 0.544^***^ |
|  | (0.194) | -/- | (0.151) |
| Security | 2.924^***^ | -/- | 0.426^***^ |
|  | (0.273) | -/- | (0.158) |
| Transport and logistics | 5.324^***^ | -/- | 0.671^***^ |
|  | (0.351) | -/- | (0.082) |
| Cleaning | 5.462^***^ | -/- | 0.036 |
|  | (1.053) | -/- | (0.114) |
| Number of observations | 7268 | 7268 | 7210 |
| Pseudo R^2^ | 0.278 | 0.042 |  |

Notes: The table shows the estimates obtained from the regression model indicated in equation (1). AMEs are the average marginal effects. Cluster-robust standard errors for 144 occupational groups in parentheses. In equation (2), the following three-digit level occupational groups of the German classification of occupations were classified as critical by Koebe et al. (2020): 343, 413, 433, 511, 512, 513, 514, 515, 516, 521, 522, 523, 524, 531, 532, 533, 541, 622, 623, 624, 633, 712, 715, 721, 723, 731, 732, 811, 812, 813, 814, 815, 816, 817, 818, 821, 824, 825, and 831. Equation (3) is based on the KRITIS list, which defines the following divisions of the German Classification of Economic Activities 2008 as critical: 1, 3, 10, 11, 21, 35, 36, 37, 38, 46, 47, 49, 50, 51, 52, 53, 58, 60, 61, 62, 63, 64, 65, 66, 84, 86, and 91. * p < 0.05, ** p < 0.01, *** p < 0.001.

Source: Working Time Survey 2019; own calculations.

Table A4: Robustness checks for working conditions in critical jobs

|  |  | Explanatory variable: Critical job (1=yes) | | |
| --- | --- | --- | --- | --- |
|  |  | Robustness check: | Robustness check: | Robustness check: |
|  | Dependent variables | Five-digit level classification of Burstedde et al. (2020) | Three-digit level classification of Koebe et al. (2020) | Economic sectors of the original KRITIS list (Pfeiffer 2020) |
| Wages | Hourly wages (log) (Coef.) | -0.021* | -0.024* | 0.012 |
|  |  | (0.009) | (0.009) | (0.030) |
| Physical proximity to others at work | Physical proximity (AME) | 0.132*** | 0.204*** | 0.054 |
|  |  | (0.011) | (0.011) | (0.048) |
|  | Home office work (AME) | -0.062*** | -0.070*** | 0.035 |
|  |  | (0.009) | (0.010) | (0.040) |
| Duration of work and atypical work hours | Weekly overtime (in hours) (Coef.) | 0.493*** | -0.140 | -0.266 |
|  |  | (0.109) | (0.111) | (0.302) |
|  | Only early or late shift work (AME) | 0.024*** | 0.013 | 0.007 |
|  |  | (0.007) | (0.007) | (0.013) |
|  | Shift work without night work (AME) | 0.025*** | 0.031*** | 0.019 |
|  |  | (0.006) | (0.006) | (0.022) |
|  | Shift work and night work (AME) | 0.034*** | 0.043*** | 0.012 |
|  |  | (0.006) | (0.006) | (0.028) |
|  | Working on Saturdays (AME) | 0.005 | -0.004 | 0.036 |
|  |  | (0.009) | (0.009) | (0.044) |
|  | Working on Saturdays and Sundays (AME) | 0.115*** | 0.071*** | -0.013 |
|  |  | (0.010) | (0.010) | (0.059) |
| Working time autonomy | Regular on-call or standby service (AME) | 0.086*** | 0.062*** | 0.049 |
|  |  | (0.008) | (0.007) | (0.026) |
|  | Make own decisions about breaks (AME) | -0.032** | -0.047*** | -0.034 |
|  |  | (0.011) | (0.012) | (0.024) |
|  | Expected to be partially accessible in private life (AME) | 0.010 | -0.000 | -0.016 |
|  |  | (0.008) | (0.009) | (0.016) |
|  | Expected to be accessible in private life (AME) | 0.040*** | 0.016 | -0.018 |
|  |  | (0.010) | (0.010) | (0.019) |
|  | Separation of work and private life possible (AME) | -0.036*** | -0.020 | 0.020 |
|  |  | (0.011) | (0.011) | (0.023) |
| Muscular and skeletal strain | Working in a standing position | 0.109*** | -0.005 | -0.124^***^ |
|  |  | (0.011) | (0.012) | (0.011) |
|  | Working in a sitting position | -0.068*** | 0.035** | 0.108^***^ |
|  |  | (0.010) | (0.011) | (0.011) |
|  | Kneeling, bending, or overhead work | 0.050*** | 0.021** | -0.044^***^ |
|  |  | (0.007) | (0.007) | (0.008) |
|  | Lifting and carrying heavy loads | 0.061*** | 0.045*** | 0.013 |
|  |  | (0.008) | (0.008) | (0.008) |
|  | Noise | 0.045*** | -0.054*** | -0.145^***^ |
|  |  | (0.010) | (0.010) | (0.010) |
|  | Bright, poor, or faint light | 0.027*** | 0.013 | 0.001 |
|  |  | (0.007) | (0.007) | (0.007) |
|  | Cold, heat, wetness, dampness, or draughts | 0.043*** | 0.013 | -0.007 |
|  |  | (0.009) | (0.009) | (0.009) |
|  | Can influence the work tasks that must be carried out | -0.013 | 0.003 | -0.024^*^ |
|  |  | (0.011) | (0.012) | (0.012) |

Notes: The table shows the estimates obtained from the regression model indicated in equation (1). AMEs are the average marginal effects. Cluster-robust standard errors for 144 occupational groups in parentheses. In equation (2), the following three-digit level occupational groups of the German classification of occupations were classified as critical by Koebe et al. (2020): 343, 413, 433, 511, 512, 513, 514, 515, 516, 521, 522, 523, 524, 531, 532, 533, 541, 622, 623, 624, 633, 712, 715, 721, 723, 731, 732, 811, 812, 813, 814, 815, 816, 817, 818, 821, 824, 825, and 831. Equation (3) is based on the KRITIS list, which defines the following divisions of the German Classification of Economic Activities 2008 as critical: 1, 3, 10, 11, 21, 35, 36, 37, 38, 46, 47, 49, 50, 51, 52, 53, 58, 60, 61, 62, 63, 64, 65, 66, 84, 86, and 91. * p < 0.05, ** p < 0.01, *** p < 0.001.
